# The association and contribution of gender-related characteristics to prevalent chronic kidney disease in women and men in a multi-ethnic population - The HELIUS study

**DOI:** 10.1186/s12889-025-22112-9

**Published:** 2025-03-04

**Authors:** Taryn G. Vosters, Frouke M. Kingma, Vianda S. Stel, Kitty J. Jager, Frans J. van Ittersum, Bert-Jan H. van den Born, Liffert Vogt, Irene G. M. van Valkengoed

**Affiliations:** 1 Amsterdam UMC, Department of Public and Occupational Health, University of Amsterdam, Amsterdam Public Health research institute, Meibergdreef 9, Amsterdam, The Netherlands; 2 Quality of Care and Ageing Later Life, Amsterdam Public Health, Amsterdam, The Netherlands; 3 Amsterdam UMC, Department of Medical Informatics, University of Amsterdam, Amsterdam Public Health research institute, Meibergdreef 9, Amsterdam, The Netherlands; 4 Amsterdam UMC, Department of Internal Medicine, Section Nephrology, University of Amsterdam, Amsterdam Cardiovascular Sciences, Meibergdreef 9, Amsterdam, The Netherlands; 5 Amsterdam UMC, Department of Internal & Vascular Medicine, University of Amsterdam, Meibergdreef 9, Amsterdam, The Netherlands; 6 Amsterdam UMC, Department of Internal Medicine, Section Nephrology, Vrije Universiteit Amsterdam, Meibergdreef 9, Amsterdam, The Netherlands

**Keywords:** CKD prevalence, HELIUS study, Gender differences, Ethnic groups

## Abstract

**Abstract:**

In chronic kidney disease (CKD), prevalence differences between sexes have been reported. While biological factors have been investigated, research on sociocultural factors is scarce. We explore the extent gender-related characteristics associate with, and contribute to, CKD prevalence in women and men in a multi-ethnic population. Cross-sectional analyses were performed on data of 12,221 women and 8,930 men aged 18–70 years across six ethnic groups from the HELIUS Study. Using age-, education-, and ethnicity adjusted Poisson regression, we determined associations between time spent on housework; primary earner status; employment status; and occupational segregation, and CKD. Population attributable fractions estimated the contribution to CKD and the extent traditional CKD risk factors explained these contributions. In women, associations with CKD were found for doing little housework, part-time work, and unemployment. In men, primary-earnership and unemployment were associated. Associations aligned across ethnic groups. Estimated contributions ranged from 1.8% for women doing little housework to 26.5% for part-time employment and 12.1% for unemployment to 37.5% for primary-earnership in men, and were hardly explained by traditional risk factors. In our study, gender-related characteristics are associated with CKD in women and men across ethnic groups. Contributions to population prevalence may hardly be explained by CKD risk factors.

**Lay Summary:**

The prevalence of chronic kidney disease (CKD) differs between women and men. We explored to what extent the risk may be associated with sociocultural expectations for women and men. We analysed data of 12,221 women and 8,930 men from six different ethnic groups. CKD was more common in all women who did little housework, worked part-time or were unemployed, and in men whose financial contribution was equal to their partners or who were unemployed. The higher risk of CKD was not explained by a higher occurrence of known risk factors. In future, specific policies or targeted interventions may be developed to reduce the risk of CKD overall and in certain population subgroups.

**Supplementary Information:**

The online version contains supplementary material available at 10.1186/s12889-025-22112-9.

## Highlights

This study found that several gender-related characteristics were associated with higher CKD prevalence, specifically low amounts of time spent on household work and working part-time in women and having an equal financial contribution and being unemployed in men. Ethnicity also influenced these results. The contribution of these characteristics to the prevalence in the population was not explained by differences in traditional risk factors. In future, specific policies or targeted interventions may be developed to reduce the risk of CKD overall and in certain population subgroups.

**Supplementary Information:**

The online version contains supplementary material available at 10.1186/s12889-025-22112-9.

## Introduction

Chronic kidney disease (CKD), classified by the cause, an estimated glomerular filtration rate (eGFR) < 60 ml/min/1.73m^2^ and/or albumin-to-creatinine ratio (ACR) ≥ 3 mg/mmol for a minimum of three months [1], is a major public health issue. CKD is more prevalent in women than men with an estimated global prevalence of 14.6% and 12.8%, respectively [2]. These sex differences are still poorly understood, fuelling a call for greater attention to possible underlying factors. Although factors related to biology and estimation of kidney function have been researched extensively, these do not sufficiently explain sex differences [3]. The relevance of sociocultural characteristics related to gender has become increasingly recognised [4–6], but studies investigating their role in CKD among women and men are scarce. The results of this exploratory study might lead to innovative and applied strategies in targeted and sex and gender-specific CKD prevention.

While sex refers to biological characteristics, gender reflects the socially constructed norms that impose and determine roles, relationships, and positional power for women and men [7]. The construct of gender depends on the context: sociocultural norms, identities, and relations change over time, and differ per cultural setting [8]. Moreover, social identities interact and interchange depending on other social dimensions, such as ethnicity, class and sexual orientation, to create differing relational levels of advantage or disadvantage [9]. Research investigating gender and gender-related characteristics as a social determinant of health has been discussed and defined in the GOING-FWD framework, in addition to previous publications [4–6].

The associations of gender-related factors with CKD have rarely been explored. One study concluded that homemaking, a traditionally female prescribed task, is associated with higher prevalence of CKD in middle- to low-income countries in comparison to people that were employed [10]. Moreover, studies including patients with end-stage renal disease showed a positive association between unemployment status [11, 12] and unfavourable renal outcomes and/or mortality. More evidence is available for cardiovascular disease (CVD), which is closely related to CKD, due to their shared risk factors, such as smoking, hypertension, and diabetes [13]. In the field of CVD associations were found between gender-related characteristics and CVD prevalence and incidence [14, 15]. Also, associations of gender-related factors have been reported for traditional CKD risk factors such as smoking [16] and diabetes [17]. Therefore, traditional CKD risk factors might be expected to mediate the relationship between gender-related characteristics and CKD. Gender-related characteristics may serve as indicators of underlying processes attributing to disease development and sex differences in disease prevalence.

This exploratory cross-sectional study will explore how gender-related characteristics are associated with CKD prevalence in women and men aged 18–70 years in a multi-ethnic population. Additionally, we estimate the potential contribution of gender-related characteristics to the CKD prevalence in women and men across several ethnic groups. Finally, we examine to what extent this contribution relates to differences in the presence of traditional risk factors, including hypertension, diabetes, obesity, hypercholesterolemia, smoking and CVD prevalence.

## Methods

### Data and study population

Cross-sectional analyses were performed on data from the Healthy Life in an Urban Setting (HELIUS) study, which was conducted amongst six ethnic groups including Dutch, African- and South Asian-Surinamese, Moroccan, Turkish and Ghanaian in Amsterdam, the Netherlands. A detailed description of the HELIUS Study has been published previously [18]. Participants (aged 18–70 years) were randomly selected from the municipal registry stratified by ethnicity, using the country of birth of the participant and their (grand-)parents. Potential participants were invited by mail, and if they did not respond, were visited by an interviewer. Of those whom we were able to contact by mail or by a home visit (55% of those invited), 50% participated in the study. Overall response rate was 28% with variations across ethnic groups. The baseline data collection took place between 2011 and 2015 via questionnaires and physical examinations at three locations. Physical examinations included, the collection of biological samples, i.e., blood and urine. Ethical approval was given by the Ethical Review Board of the Amsterdam University Medical Centre.

The proposed study concerns participants who completed the questionnaire and physical examination at baseline (*n* = 22 162). Participants with Javanese, other/unknown Surinamese or unknown/other ethnic background origin were excluded due to small sample sizes (*n* = 548). Participants were also excluded if data was not available for CKD (i.e., eGFR and ACR, *n* = 184), at least one gender-related characteristic (*n* = 45) or covariates (*n* = 234). This leaves a total sample of 21,151 participants, 12,221 women and 8,930 men (Supplementary figure S1).

### Gender-related characteristics (independent variables)

Four gender-related characteristics were chosen, as done previously by our research group [14, 15, 17] based on the work of Pelletier et al. (2015) [5], Johnson et al. (2009) [4] and aligning with the GOING-FWD framework [6] and availability in the questionnaire: (1) Time spent on housework; (2) Employment status; (3) Primary earner status; (4) Working in a female-/male-dominated occupation (Supplementary Figure S2 & Supplementary table S1). The variables can change over time and are traditionally associated with social expectations or norms ascribed to women and men in western society, that may potentially serve as indicators of underlying processes [6, 19]. Although gender roles and expectations are no longer as strict as they may have been decades ago, it was shown that these traditional western gender roles are still attributed to women and men in Dutch society [20, 21].

### Chronic kidney disease (dependent variables)

CKD was defined according to the 2012 ‘Kidney Disease: Improving Global Outcomes’ (KDIGO) Clinical Practice Guideline. The Glomerular Filtration Rate was estimated (eGFR) with the CKD Epidemiology (CKD-EPI) equation using creatinine obtained from a fasting venous blood sample. Albuminuria was assessed by measuring the albumin-to-creatinine ratio (ACR) in a first morning urine sample and plasma creatinine concentration was determined by a kinetic colorimetric spectrophotometric enzyme assay (Roche Diagnostics, Japan). Both measures were based on a single sample. Kinetic spectrophotometric and immune chemical turbidimetric methods were used to analyse urine creatinine and albumin concentration. Prevalent CKD was defined as eGFR < 60 ml/min/1.73m^2^ and/or ACR ≥ 3 mg/mmol [22]. Secondary outcomes were 1) eGFR < 60 ml/min/1.73m^2^ (Yes/No) regardless of ACR and 2) ACR > 3 mg/mmol (Yes/No) regardless of eGFR.

A correction for ethnicity in participants with a Ghanaian or African-Surinamese background is included in the CKD-EPI 2012 equation, used for the primary outcome. However there has been discussion regarding its use, therefore sensitivity analyses included the CKD-EPI 2021 equation which does not correct for ethnicity, for comparison [23].

### Other variables

Other variables included occupational level, household composition, age, educational level, ethnicity, and sex. These were considered as confounders and potential effect modifiers in the case of ethnicity and sex. The following variables were defined as risk factors for CKD by international guidelines [22]: hypertension, smoking status, hypercholesterolemia, diabetes mellitus, obesity and CVD, and were included as potential mediators (Supplementary Figure S2 and Supplementary Table S1).

### Statistical analyses

First, characteristics were described in women and men in the total sample and within ethnic groups. This was done by percentages and number of observations for categorical variables, means and standard deviations for normally distributed continuous data and medians and interquartile ranges for non-normal distributions (evaluated via visual inspection (frequency and QQ plots)).

The potential associations between the gender-related characteristics and CKD prevalence were determined using Poisson regression with a robust variance estimate. Analyses were done separately for women and men, considering that sex and gender-related characteristics could interact with one another [4]. The analyses were adjusted for confounders based on our conceptual model (Supplementary Fig. 1); age (model 1), then additionally for ethnicity and educational level (model 2, main model), and reported as prevalence ratios (PR’s). An interaction term was added between each of the gender-related characteristics and sex in model 2 to assess whether associations differed by sex. In addition, the results for model 2 were stratified by ethnicity, rather than adjusted for, to see whether the patterns of association were similar across ethnic groups, as gender-related roles and behaviours may differ by ethnic group or culture and their intersections.

To estimate the contribution of gender-related characteristics to prevalent CKD in women and men in the population, the population attributable fraction (PAF) was calculated for characteristics for which a significant association was observed in model 2 [24]. This method assumes gender-related characteristics are modifiable and that there is a causal relationship between the dependent and independent variables, however we recognise we cannot prove such with cross-sectional analyses. We dichotomised the gender-related characteristics into ‘non-exposed’ and ‘exposed’ groups where possible, by creating new binary variables. In cases where no clear trend was evident, a multi-categorical calculation was used. The exposed group was the group for which we observed a statistically significantly higher prevalence of CKD in the former step. This implies that for variables with negative associations, the reference category was changed. The adjusted PAF for bi-category $\left( {PAF = pd\frac{{PR - 1}}{{PR}}} \right)$ and multi-categorical $\left( {PAF = 1 - \sum {\frac{{pd}}{{PR}}} } \right)$ estimation was used because it produces internally valid estimates even in the case of confounding [24]. Multi-category variables a single statistically significantly associated category were transformed into a binary variable to avoid potentially overestimating the contribution when considering non-significant estimates for other categories. *Pd* refers to the proportion of cases exposed to the risk factor. To estimate to what extent the contribution is mediated by traditional risk factors we repeated the analysis using the PR estimates from models including risk factors hypertension, diabetes, obesity, hypercholesterolemia, smoking status and CVD prevalence as mediators (model 3). Contributions were further estimated for ethnic groups, using the PRs derived from the total sample for women and men, as some groups were too small for group-specific PR estimates.

Finally, sensitivity analyses included repeating the main analyses with eGFR < 60 ml/min/1.73m^2^ or only ACR ≥ 3 mg/mmol as outcome variables, to investigate whether the associations were being driven by only one or both of the measures considering these measure different aspects of kidney function. As well as restricting the main analyses for time spent on housework and primary earner status to adults who previously stated to live with other adults. In this group the prevalence gender-related roles or behaviours may be overestimated, as housework and income may not be shared. Meanwhile employment status and occupational segregation was restricted to all those who have a job. Furthermore, analyses explored whether the adjustment for additional occupational variables occupational level and employment status for occupational segregation changed the interpretation for the results. This was done because the female-/male ratio of an occupation is associated with the occupational level, and because reporting of (past) occupation may be affected by current work status. Lastly, it was explored whether the results of the correction for ethnicity in the CKD-EPI 2012 estimation of eGFR differed from the ethnicity-free CKD-EPI 2021 estimation, to determine whether our choice of algorithm affected our results.

All analyses were done using IBM SPSS Statistics (V. 28; [25]). A p-value of < 0.05 was considered statistically significant.

## Results

The study sample consisted of 57.7% women and 42.2% men, for whom median age was 43.8 and 44.9 years respectively (Table 1). Amongst women and men, the majority attained a medium-high to high educational level and lived with another adult. Men had higher rates of traditional risk factors for CKD than women.

Except for Primary earner status, the distribution of gender-related characteristics among women and men in the total sample largely corresponded with their traditional gender role expectations. In contrast to the overall population, African-Surinamese women had a higher percentage of primary earners over men (Supplementary Table S2) and Ghanaian men were more likely to work in more female dominated occupations.

In women, overall CKD prevalence was 6.3% versus 5.4% in men (Table 1). The observed pattern of difference between women and men was similar across African-Surinamese, Turkish and Moroccan groups (Supplementary Table S2). Estimated prevalence was slightly higher in men than women in Dutch, Ghanaian and South Asian-Surinamese subgroups. The total sample prevalence of eGFR < 60 ml/min/1.73m^2^ was 1.4% in men and 1.1% in women and the prevalence of ACR ≥ 3 mg/mmol was 4.6% and 5.5% in men and women, respectively. The pattern of the secondary CKD outcomes in women and men corresponded across ethnic groups. Additional analyses using the ethnicity free CKD-EPI 2021 equation revealed similar prevalence patterns of CKD in the total population and higher CKD prevalence estimates in most ethnic groups (Table 1 & Supplementary Table S2).

**Table 1:** Baseline characteristics in the total population

		Women	Men
		***n*** ** = 12,221**	***n*** ** = 8,930**
**Age (years)**		43.8 (13.1)	44.9 (13.2)
**Ethnicity**	*Dutch*	19.8	22.8
	*South Asian-Surinamese*	13.4	15.1
	*African-Surinamese*	20.2	17.7
	*Ghanaian*	11.4	10.0
	*Turkish*	15.8	17.9
	*Moroccan*	19.4	16.6
**Educational Level (missing ** ***n*** ** = 112)**	*Low*	20.5	14.1
	*Medium-Low*	24.4	28.8
	*Medium-High*	28.7	29.6
	*High*	26.3	27.5
**Occupational Level (missing ** ***n*** ** = 189)**	*Elementary*	14.4	12.4
	*Low*	20.3	32.2
	*Intermediate*	23.4	21.1
	*High or Academic*	22.4	24.9
	*Not applicable*	19.5	9.4
**Household composition**	*Living with > 1 adults*	70.1	73.4
	*Living without adults*	29.9	26.6
**Hypertension (missing ** ***n*** ** = 27)**	*Yes*	29.6	36.7
**Smoking Status (missing ** ***n*** ** = 55)**	*Yes*	18.1	32.1
**Hypercholesterolemia (missing ** ***n*** ** = 8)**	*Yes*	18.6	21.7
**Diabetes Mellitus**	*Yes*	8.4	10.4
**CVD Prevalence (missing ** ***n*** ** = 203)**	*Yes*	4.1	7.0
**Obesity (missing ** ***n*** ** = 14)**	*Normal*	37.6	40.2
	*Overweight*	31.3	42.5
	*Obese*	31.1	17.4
**Time spent on Housework**	*0–3 h/week*	15.5	40.9
	*> 3–7.75 h/week*	21.0	27.1
	*> 7.75–16 h/week*	29.3	22.1
	*> 16 h/week*	34.1	9.9
**Primary Earner Status (missing ** ***n*** ** = 241)**	*Yes*	50.0	70.7
	*Equal income*	14.9	13.8
	*No*	35.1	15.5
**Employment Status (missing ** ***n*** ** = 189)**	*Fulltime (> 32 h/week)*	30.5	56.8
	*12–32 h/week*	21.2	10.3
	*< 12 h/week Incl. Homemakers*	15.4	2.3
	Not employed*	32.9	30.5
**Female-/Male-dominated occupation (missing ** ***n*** ** = 1778)**	*< 25% female workers*	3.9	34.4
	*26-50% female workers*	17.8	32.3
	*51-75% female workers*	36.4	22.4
	*> 76% female workers*	25.2	3.7
	Not applicable**	16.7	7.1
**CKD Prevalence (CKD-EPI 2012)**	*Yes*	6.3	5.4
**eGFR < 60 ml/min/1.73 m** ^**2**^	*Yes*	1.1	1.4
**ACR** **≥** **3 mg/mmol**	*Yes*	5.5	4.6
**CKD Prevalence (CKD-EPI 2021)**	*Yes*	6.3	5.5

Data are presented as percentages. CKD = Chronic kidney disease; CVD = cardiovascular disease; eGFR = estimated glomerular filtration rate; ACR = albumin-to-creatinine ratio; *unemployed, pensioners, students, welfare recipients; ** not applicable to students, pensioners, and those unable to work

### Association of gender-related characteristics with CKD

**Fig. 1:**
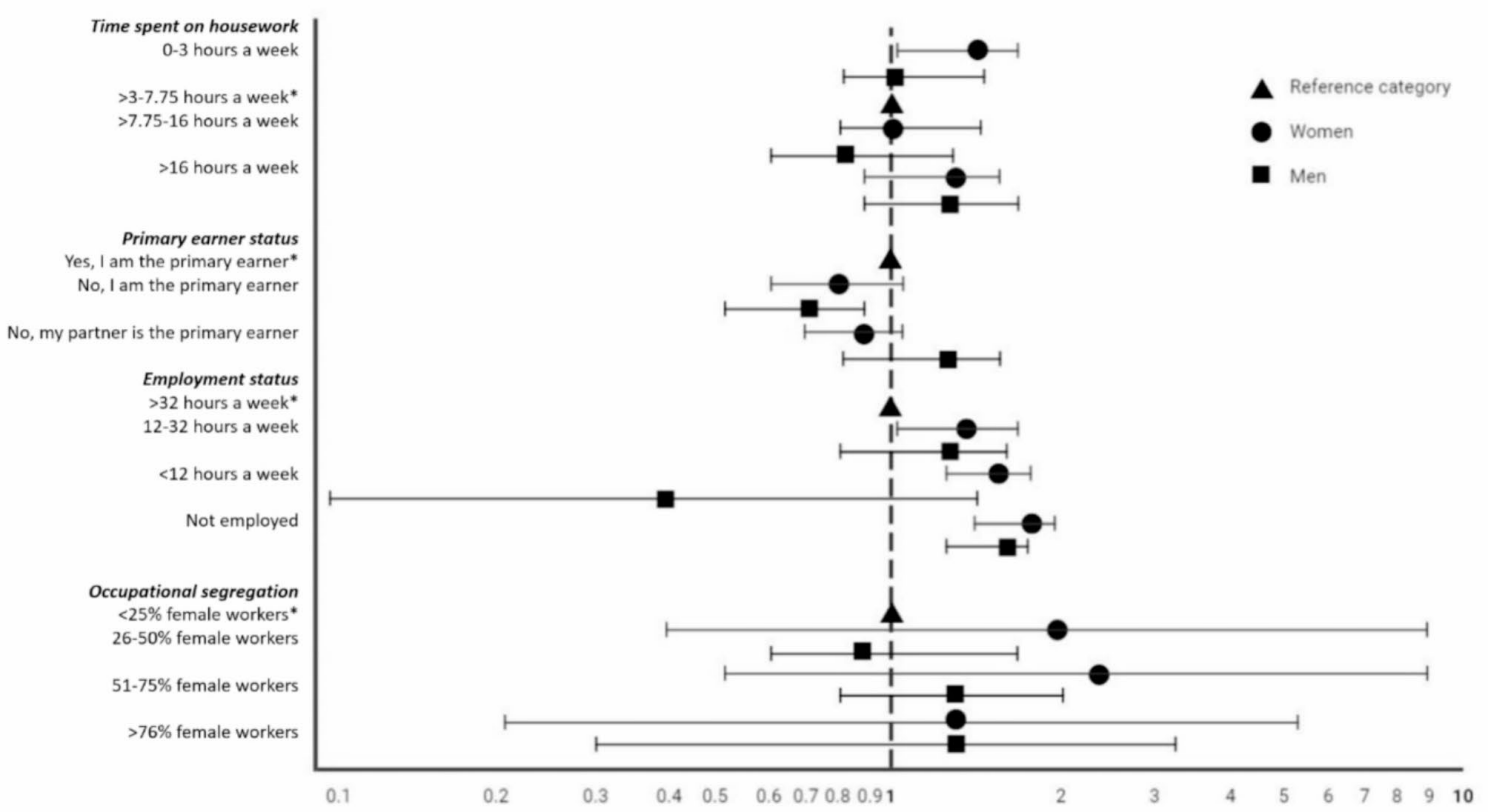
Association between gender-related characteristics and CKD in the total population. Associations for Model 2: adjusted for age, education and ethnicity; Shapes = prevalence ratios estimated by Poisson regression; Bars = 95% confidence intervals; *reference category; for other remaining models see supplementary Table 3

Several gender-related characteristics were associated with CKD and its secondary outcome measures (Fig. 1, Supplementary table S3-5). The most feminine and most masculine categories of housework were associated positively with CKD prevalence in women, depicting a U-shaped association. However, the association only remained for the most masculine category (0–3 h/week) in the main model. In men, particularly those reporting 0–3 h to household work had a lower prevalence of CKD compared to women (p for interaction household work*sex = 0.011). Estimates for ACR ≥ 3 mg/mml, but not eGFR < 60 ml/min/1.73m^2^, showed a similar direction.

Compared to being the primary earner, having an equal contribution to household income showed a negative association with having CKD in men only (PR = 0.703 [0.52–0.95]). Estimated associations for eGFR < 60 ml/min/1.73m^2^ and ACR ≥ 3 mg/mml were in similar directions. In women, not being the primary earner was associated with an eGFR < 60 ml/min/1.73m^2^, but not with ACR ≥ 3 mg/mml. The associations remained in the analyses restricted to those who lived with other adults (Supplementary table S6).

In women, working part-time or being unemployed had a higher prevalence of CKD compared to those working fulltime (Supplementary Table S3-5). Men who were not employed had a higher prevalence of CKD compared to those working fulltime (PR = 1.426 [1.17–1.75]) (Fig. 1 (main model), Supplementary Table S3-5 (all models)). The direction of estimates was similar for the prevalence of eGFR < 60 ml/min/1.73m^2^ and corresponding significant associations were found for prevalent ACR ≥ 3 mg/mml. Working in a female-/male-dominated occupation did not show consistent associations for prevalent CKD in women or men. Further sensitivity analyses did not change our interpretation of the findings (Supplementary Table S6).

After stratifying by ethnicity, the association patterns for CKD in women and men remained largely similar, with a few exceptions, e.g., in Dutch women performing high and low amounts of housework were associated with CKD, as opposed to only low amounts in the total sample and in South Asian-Surinamese women estimations for all categories of housework and employment status were reversed compared to the total sample (Supplementary Table S7-12).

### Contribution gender-related characteristics to CKD

**Fig. 2:**
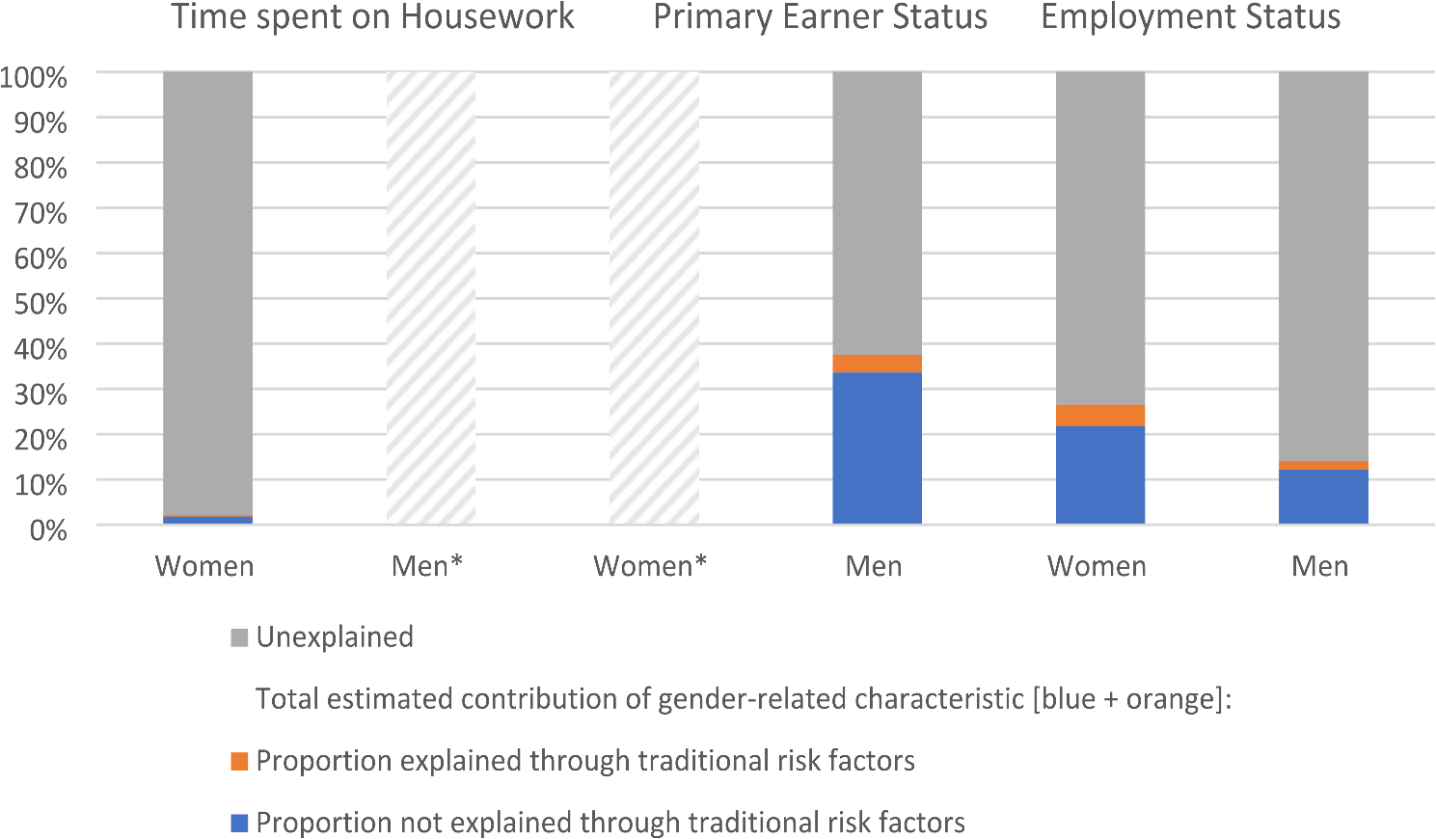
Estimated contribution to CKD - population attributable fraction. Time spent on housework was dichotomised into high amounts (> 7.76 h per week) vs. low to no amounts (< 7.75 h per week, exposed category); primary earner status could not be dichotomised due to irregular association patterns, therefore multicategorical estimations were made; Employment status in women was dichotomised into working fulltime (> 32 h per week) vs. working partime/not working (0–31 h per week) and for men it was dichotomised into working and not working (unemployment); * No PAFs were calculated due to non-significant association between the gender-related characteristic and prevalent CKD

In women, the contribution of the gender-related characteristics to the prevalence of CKD in the population varied from 2.1% for doing little to no amounts of housework to 26.5% for being part-time employed (Fig. 2, Supplementary Table S13).

The traditional risk factors hardly explain the contributions (Supplementary table S13). The association estimates for model 3 adjusting for the traditional risk factors were similar to model 2 (Supplementary table S3-5). After adding the risk factors, the estimated contribution of CKD of the variables attenuated to 1.8% for little amounts of housework and 21.8% for part-time or unemployment status. Similarly, the multi-categorical estimation for primary earner status revealed a contribution of 37.5% and 33.6% after the inclusion of risk factors. For the additional CKD outcomes, the contribution of all gender-related characteristics were also hardly or not explained by the presence of conventional CKD risk factors. The estimated contributions of the gender-related characteristics to prevalent CKD across ethnic groups largely corresponded to the contributions of women and men in the total sample, with a few variations (Supplementary table S14). For instance, employment status contributed more to CKD in Turkish and Moroccan women compared to women in the total population.

## Discussion

Gender-related characteristics, specifically low amounts of time spent on housework and part-time employment in women and having an unequal primary earner distribution, or unemployment status in men were found to be significantly associated with a higher prevalence of CKD. Gender-related characteristics contributed to between 1.8 and 37.5% of prevalent CKD in the population, and this was only minimally explained through mediating risk factors, i.e. smoking, hypertension, diabetes mellitus, hypercholesterolemia, obesity, and CVD prevalence. Although the power was limited to calculate associations for individual ethnic groups, patterns of associations and contributions seemed consistent.

Prior to discussing our findings, we note some limitations. First, due to the exploratory and cross-sectional nature of the study we are able to identify associations which is valuable in a multi-ethnic setting. Therefore, we cannot make conclusions on any causal relationships, as we cannot be certain that these associations and estimated contributions reflect an effect of the gender-related factors and mediators on CKD. CKD may affect participants’ physical ability, hindering them from carrying out tasks and responsibilities, both personally and professionally, which could result in reverse causation. This is also reflected in the PAF methodology, as it assumes a causal relationship between the variables. A longitudinal cohort study may provide more precise and reliable results. Second, the outcome measurements were based on a single sample, although in clinical practice repeat measurement is recommended. Moreover, we used the GFR estimate that included a factor for ethnicity (CKD-EPI 2012). Exclusion of this factor in the CKD-EPI 2021 equation, as recommended by Inker (2022) [26], did not change the overall prevalence and showed similar association patterns overall, but expectedly estimated a higher CKD prevalence for various ethnic subgroups. Others have recommended the use of an algorithm that takes Cystatin C into account, because it is less likely to be influenced by dietary habits and muscle mass than creatinine based measurements and it eliminates the consideration of ethnicity and sex altogether when estimating CKD [26, 27]. This could not be done in our study, thus our CKD estimate could not be as accurate for both sexes and for certain subgroups resulting in a possible bias in the comparisons across groups.

Furthermore, our measurement of gender-related characteristics may have underestimated the contribution of gender. We did not cover all domains of gender, and may not have measured all relevant aspects of the included domains. For instance, previous studies have shown that factors such as care-giving and risk-taking behaviours may also affect health [5]. A validated questionnaire was not available to measure gender-related characteristics at the time of data collection. Although the selection of variables was carefully planned, the chosen proxies were not included specifically to measure gender-related characteristics in the HELIUS study. For example, housework was given in hours, rather than the distribution of housework among those living in the household. Additionally, we note that answers could be subject to social desirability and questions in the survey may be interpreted or valued differently by women and men and across subgroups, resulting in participants potentially not answering truthfully due to social expectations. Simultaneously, assigning western gender roles to ethnic groups that may have varying gender ideals, could cause unreliable results. Gender ideals are influenced by contrasting social, cultural, and religious considerations [4]. However, the distribution of feminine and masculine characteristics largely aligned across most groups, with only some exceptions. As there is very limited research in this field, we believe that our results are still of value considering the multi-ethnic setting, showing the relevance of further research within this field with many of the above limitations to be overcome within this further research.

### Discussion of main findings

Our findings indicate that gender-related characteristics associate with CKD in addition to biological sex and traditional risk factors. In particular low amounts of housework, primary earner status, part-time employment and unemployment were associated. Our study was the first to investigate multiple gender-related characteristics in relation to CKD. Previous work in population based studies had only been done on single characteristics, such as being unemployed [11, 28] and housework and prevalent CKD [10], and studies with other non-communicable chronic diseases including prevalent and incident CVD [14, 15] and prevalent and incident diabetes mellitus [17]. Fewer associations with gender-related characteristics were found for lower eGFR and ACR > 3 mg/mml the secondary CKD outcomes than the primary CKD outcome, however the directions of association were similar. This could be due to smaller numbers of events, limiting the power of the analyses.

As in previous cross sectional and prospective studies on other outcomes [14, 15, 17], we found that associations for some of the gender-related characteristics differed between men and women, but remained largely consistent across ethnic groups. Uniform “masculine” or “feminine” disease pathways in either women or men cannot be determined, due to the varied nature of the results. The differences in the direction of associations between characteristics aligns with the fact that the selected gender-related characteristics were found to only weakly correlate previously [14], and suggests that the different characteristics measure different aspects of social structures related to health. Nevertheless, work on CVD incidence and diabetes prevalence in the same population has suggested a more “feminine” pathway in women. More work is needed to unravel whether these differences indeed indicate that various characteristics and other aspects of social structures may relate differently to health outcomes, potentially through different mechanisms, or whether findings reflect methodological choices in the abovementioned studies.

We expected to find a correlation between occupations that are typically carried out by women or men and CKD due to sedentary work or toxin exposure [4, 29], however no associations with CKD were found for occupational segregation for women or men. These results align with previous work done with this variable in association with diabetes prevalence and incidence, estimated CVD risk and incidence of CVD [14, 15, 17]. Associations differed by sex for housework. In women, however not in men, doing low amounts of housework, the most masculine category, was positively associated with CKD prevalence. This is consistent with previous work [10, 14, 15]. The results of the present study are similar to those of Bolijn et al. (2021) in HELIUS [15], which show women doing moderate amounts of housework had lower CVD hazards than women doing low amounts of housework per week. That study suggested a U-shaped association, also reporting a higher risk for the most feminine category of large amount of time spent doing housework. Women who dedicate a large amount of time to doing housework, are likely to be homemakers. Khajehdehi et al. have shown that homemaking is associated with CKD [10]. A cross-sectional population based study in Spain showed that homemakers tend to have a lower psychosocial wellbeing which can lead to poorer health, including chronic illnesses [30]. In our study, the prevalence for those in the highest category of household work was, however, not significantly different from those doing moderate amounts of housework in the main model.

In men, the most masculine category of being a primary earner was associated with CKD prevalence. Being a primary earner causes stress and pressure to provide for the household, which may explain the association with CKD as stress is a known risk factor for chronic illnesses and metabolic deregulation [31–33]. Moreover, the findings suggest that men who are not primary earners also have an increased risk of CKD compared to equal earners. Importantly, sensitivity analyses in those that are able to work, show that the association was not due to CKD or other chronic illnesses already being present hindering men from working and being the primary earner. Not having financial autonomy could increase the risk of developing CKD because access to healthcare could be impacted, leading to late diagnosis and little to no use of prevention measures [31, 33, 34]. Moreover, it could be speculated that men not being able to fulfil the societal expectations of taking on certain financial responsibilities, could lead to stress-related symptoms for not meeting this expectation, which in turn could increase the risk of developing CKD.

In women and men, not being employed or part-time employment (in women only) was associated with prevalent CKD. The findings were in line with a previous population-based study [11], although the authors did not differentiate between sex, showing unemployment is a risk factor for CKD. Unemployment has been shown to have negative effects on individuals’ health, increasing the risk of chronic illnesses through various pathways [14, 15, 33, 35]. Studies show this could be explained by low health literacy levels [36], as well as prevalent income inequalities [37]. Women working part-time additionally tend to be impacted by the ‘double burden’ of household duties and financial responsibilities [31].

Aside from occupational variables being precise gender indicators, they are likely to be swayed by the health worker effect [38], claiming those in better health are more likely to be represented as fulltime employees than sick and handicapped workers. Furthermore, the group of unemployed participants included pensioners, who due to their age have a higher risk of developing CKD [39]. Additional analyses however showed this association remained after excluding pensioners, students and those unable to work.

The results of this study suggest roles and behaviours traditionally assigned to both genders could directly and indirectly contribute to prevalent CKD in the population, in some cases up to 52%. Khajehdehi et al. hypothesised in their cross-sectional study that an increased risk of CKD with gender-related factors could be related to pre-existing traditional risk factors (age, obesity, diabetes mellitus and hypertension) but did not run these analyses [10]. However, in our data the inclusion of traditional risk factors hardly explained the associations and the contribution of the characteristics to CKD, suggesting that other mechanisms may play a role. Although the traditional risk factors capture some of the possible lifestyle-related factors (e.g. obesity relating to physical activity and diet), there may also be others such as environmental factors, intake of nephrotoxic herbal medications, and alcohol intake which could be influenced by gender [40–42]. Moreover, other factors could contribute to the prevalence of CKD such as depression and stress. For instance, unemployment, could result in higher rates of depressive symptoms [42], potentially also affecting the hypothalamic-pituitary-adrenocortical axis, and immune system. These interactions and potential alternative disease pathways require a more in-depth investigation, which we were not able to combine into this study.

If our findings are confirmed, the implications of this exploratory study could be the development and evaluation of targeted interventions, e.g., for unemployed men or women working part-time. Specific policies or interventions may be developed and introduced to target risk factors underlying the higher risk for CKD identified in future work in these groups.

In conclusion, in this study we investigated the contribution of several gender-related characteristics to CKD in both women and men in a multi-ethnic population. Our findings suggest that several gender-related characteristics, specifically housework, household financial contribution and one’s employment status, are associated with CKD. Moreover, the results suggest that these gender-related characteristics contribute to the prevalence of CKD in women and men across several ethnic groups, and this contribution is hardly explained by traditional risk factors.

## Electronic supplementary material

Below is the link to the electronic supplementary material.

Supplementary Material 1

## Data Availability

Data may be obtained from a third party and are not publicly available. The HELIUS data are owned by the Academic Medical Center (AMC) in Amsterdam, the Netherlands. Any researcher can request the data by submitting a proposal to the HELIUS Executive Board as outlined at http://www.heliusstudy.nl/en/researchers/collaboration. Requests for further information and proposals can be submitted to heliuscoordinator@amsterdamumc.nl. The HELIUS Executive Board will check proposals for compatibility with the general objectives, ethical approvals and informed consent forms of the HELIUS study, and potential overlap with ongoing work affiliated with HELIUS. There are no other restrictions to obtaining the data and all data requests will be processed in the same manner.
