# Supplementary material for: The association and contribution of gender-related characteristics to prevalent chronic kidney disease in women and men in a multi-ethnic population - The HELIUS study
Source: BMC Public Health. 2025 Mar 4;25:853. doi: 10.1186/s12889-025-22112-9 (PMC11877908; doi:10.1186/s12889-025-22112-9)
Supplement: Supplementary file 1 — Supplementary Material 1 [file 12889_2025_22112_MOESM1_ESM.docx]

Supplementary Materials

Supplementary Figure 1: Flowchart of participant selection Supplementary Figure 2: Simplified conceptual model


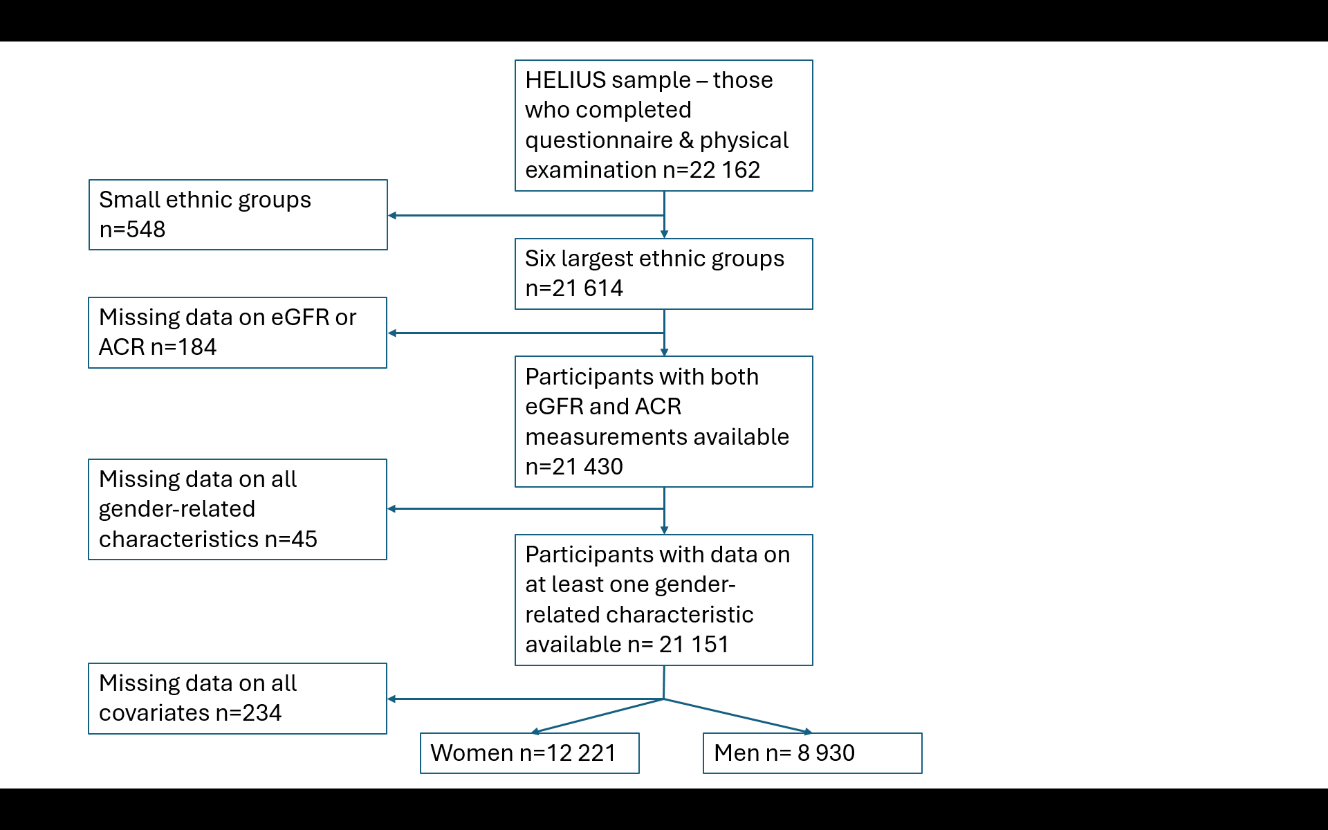

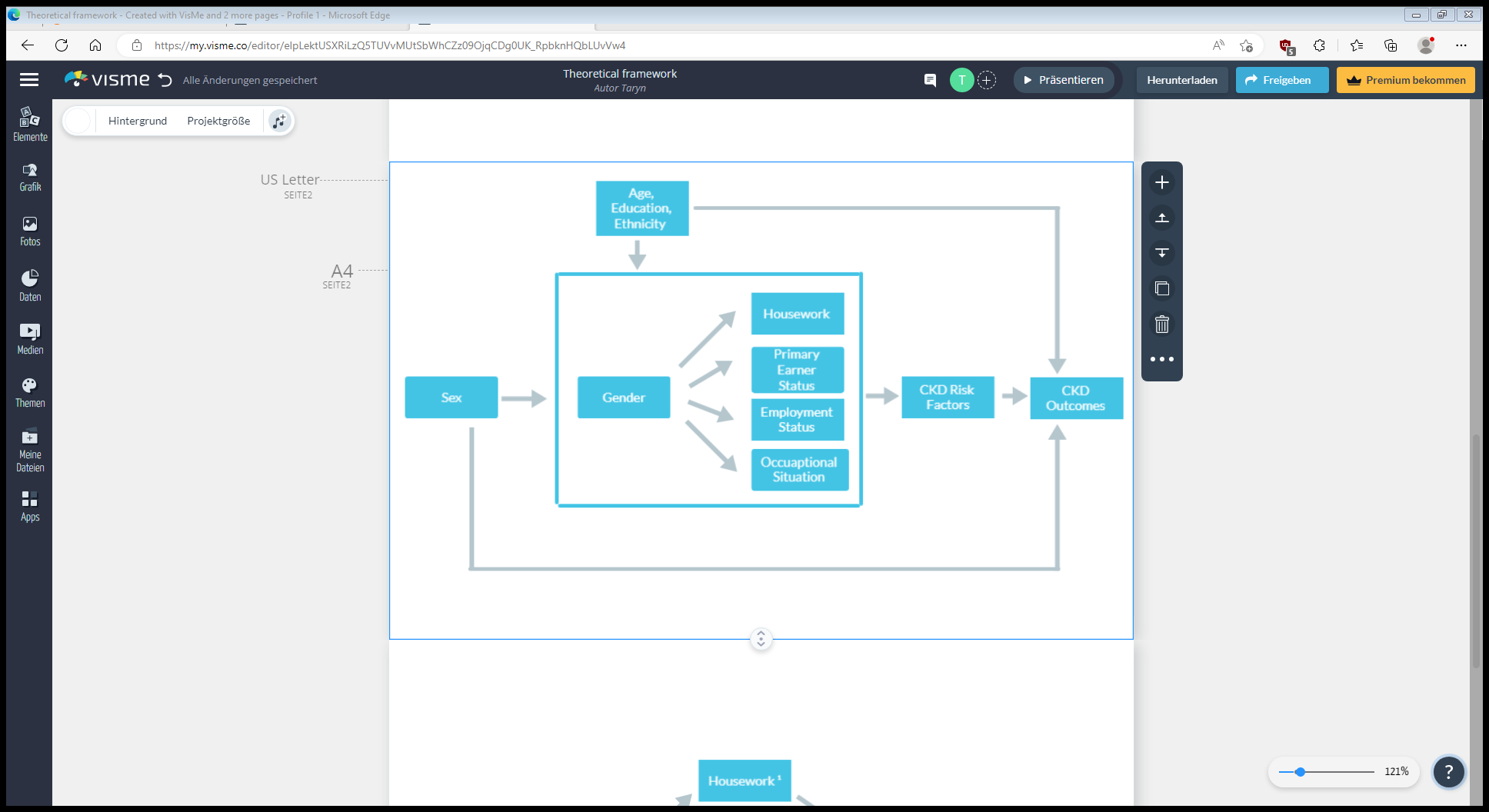


*CKD Risk Factors: Hypertension, Diabetes mellitus, smoking status, hypercholesterolemia, Obesity; CKD Outcomes: Prevalence based on eGFR and ACR values; Sensitivity analyses included the adjustment of occupational level and employment status for the association analysis between Occupational Segregation and prevalent CKD.*

*Supplementary figure 3: Sensitivity Analyses in the overall population*

*
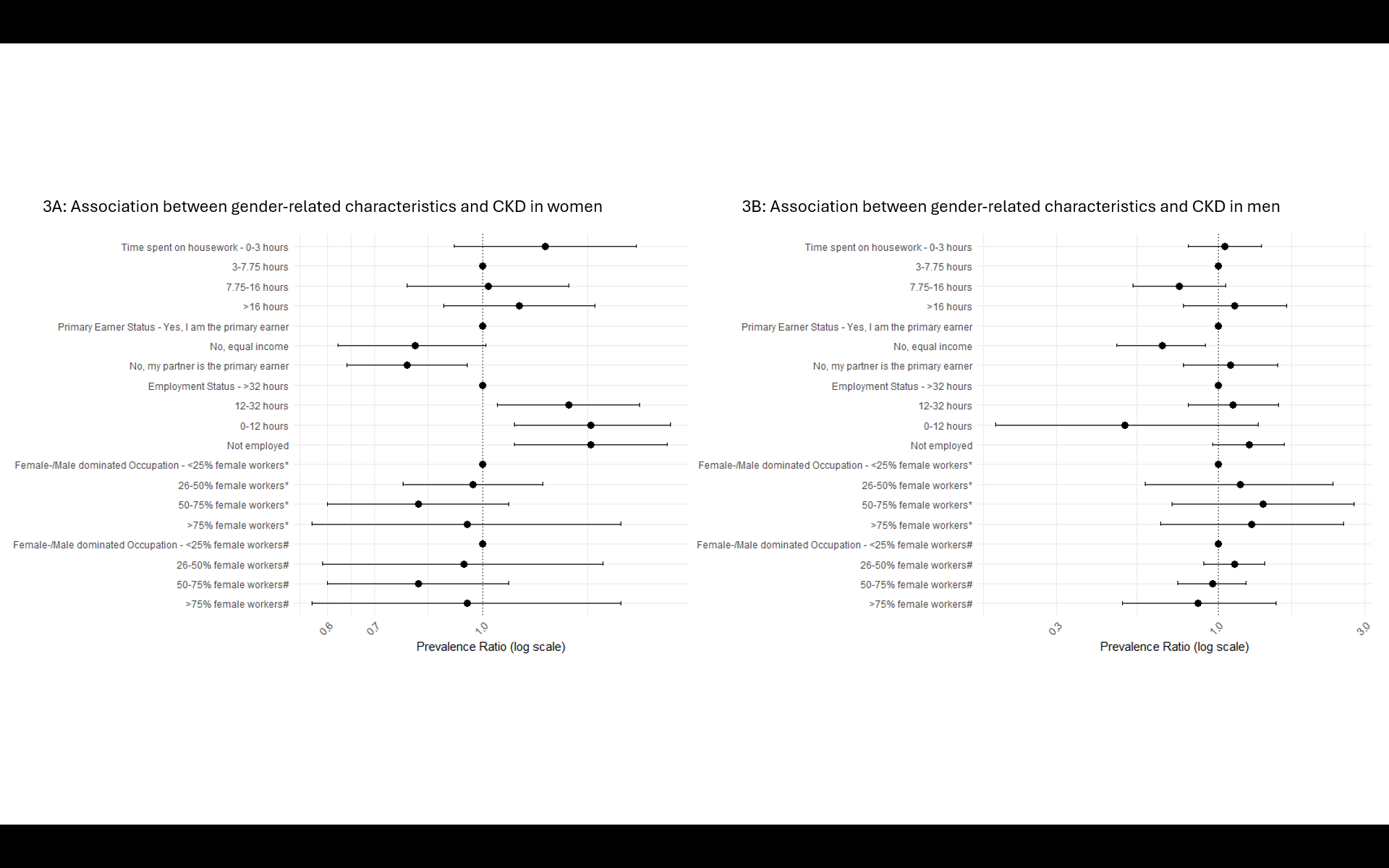
*

*Main model (Adjusted for Age, Ethnicity and Educational level ) analyses with sample restriction methods defined in the sample; Time spent on housework - restricted to those living with another adult(s);* *Primary Earner Status - restricted to those living with another adult(s);* *Employment Status - excluding students, pensioners, and those unable to work; Female-/Male dominated occupation - ** *Excluding students, pensioners, and those unable to work, ^#^ Model 2 + Employment Status & Occupational level; figure depicts prevalence ratios and their corresponding 95% confidence intervals - see below supplementary table 6 for estimates*

*Supplementary figure 4:* *Sensitivity Analyses using CKD-EPI 2021 equation*

*
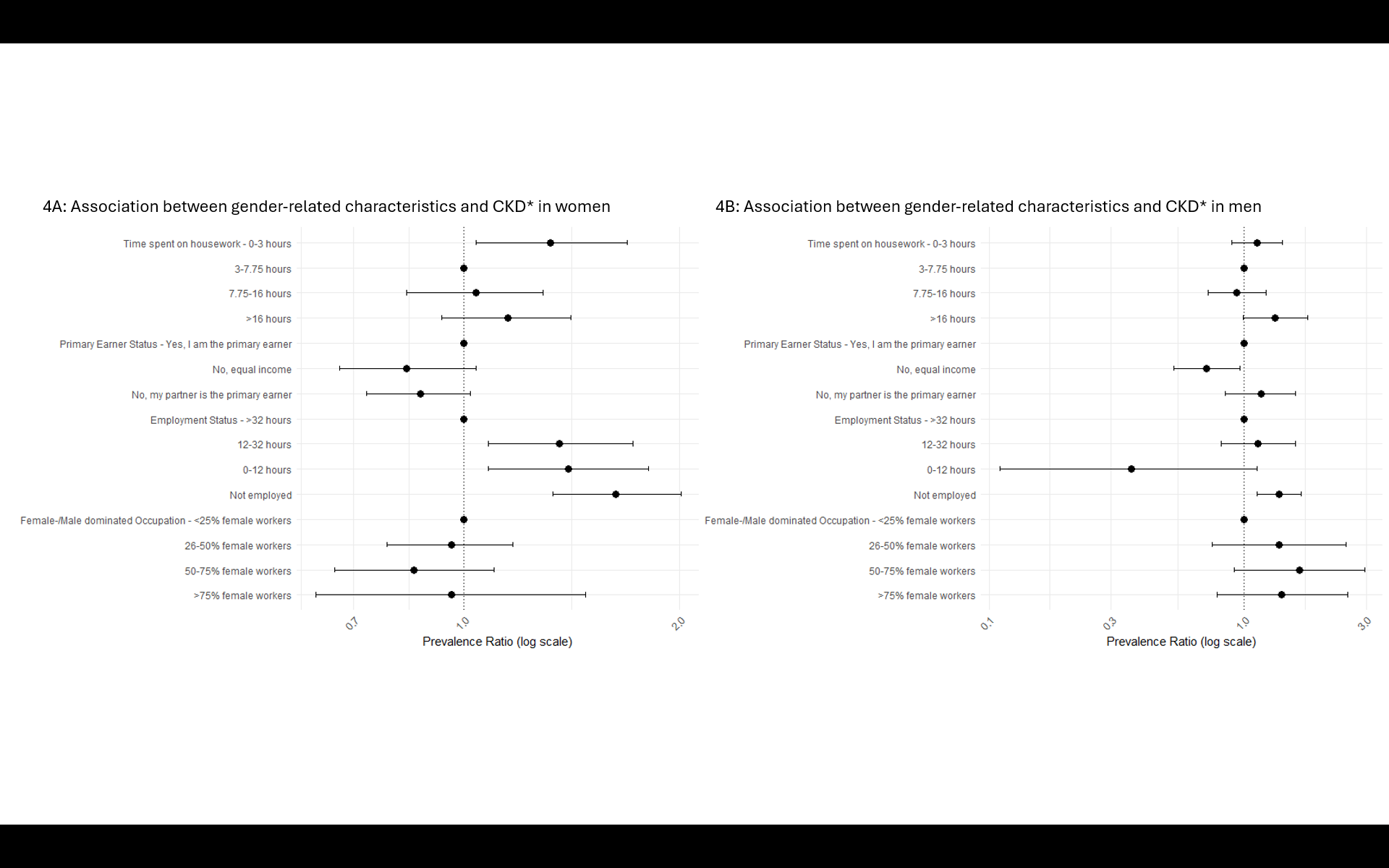
*

*Main model analyses using the *CKD-EPI 2021 ethnicity free equation rather than the CKD-EPI 2012 equation, see below supplementary table 6 for estimates*

*Supplementary table 1: Description of variable definitions*

| Variable | Description |
| --- | --- |
| Gender-related characteristics | |
| Time Spent on Housework | Time spent on Housework was based on the total number of hours per week dedicated to light/moderate (e.g., cooking, doing dishes, feeding children, vacuum cleaning, or grocery shopping ) or heavy household work (e.g., scrubbing floors, beating carpets, carrying heavy groceries ) based on a normal/standard week the last few months. This variable was classified into quartiles (0-3 hours/week; >3-7.75 hours/week (reference group); >7.75-16 hours/week; >16 hours/week ), where being categorised into the lowest quartile was regarded as most masculine, whereas being categorised into the highest quartile was regarded as most feminine, due to women being expected to take on most of the housework (1). |
| Primary Earner Status | Primary Earner Status was based on the question of who is currently the main breadwinner in the household. Answers were classified into three groups: yes, I am the primary earner; equal income; and no, I am not the primary earner. Since carrying the financial responsibility of the household is still traditionally the male’s role (2) and statistically they are overrepresented with this regard (3), being the primary earner of the household income was regarded as most masculine, while not being the primary earner of the household income was regarded as most feminine. Having an equal income was regarded as neither masculine, nor feminine. |
| Employment Status | Employment Status was based on number of paid work hours per week at the time of survey and was classified into four groups: fulltime (≥32 hours per week ), part time (12-32 hours per week), fulltime homemaker (including those who perform paid work <12 hours per week), and not employed (pensioners, students, unemployed workers/jobseekers, persons unable to work, welfare recipients). Since women are more likely to work part time, as opposed to men who tend to work fulltime (2,4), being fulltime employed was regarded as most masculine, whereas being a fulltime homemaker was regarded as most feminine. The group which was ‘not employed’ was regarded as neither masculine, nor feminine. |
| Working in female-/male dominated occupation (Occupational segregation) | Occupational Segregation reflected both past or current occupations. For Working in a female- or male-dominated occupation, four categories were created based on the most recent numbers at baseline of men and women per occupation category (occupations with ≤25% female workers, 26-50% female workers, 51-75% female workers, and ≥76% female workers) according to the Central Bureau for Statistics Netherlands. The first category was regarded as ‘male-dominated occupation’ and most masculine based on the lack of female representation within the work environment, whereas the fourth category was regarded as ‘female-dominated occupation’ and most feminine, based on the variable ‘fulltime’ or ‘part-time’ employment. |
| Other Variables | |
| Occupational level | Occupational level was classified according to the Dutch Standard Occupational Classification system (5) which provides an extensive systematic list of all professions in the Dutch system. Occupational level consisted of five categories, based on job title and job description, including a question on fulfilling an executive function. |
| Household composition | Household composition was defined as living with or without one or more adults (categorical), self-reported during the baseline questionnaire. |
| Age | Age was measured in years (continuous), based on the age at the time of baseline questionnaire. |
| Educational level | Educational level was defined as highest educational level attained either in the Netherlands, or in the country of origin, categorized as in low, medium-low, medium-high, and high (categorical, self-reported). Educational level has been proven to be a more precise indicator of SES in the Netherlands than other proxies such as income and occupational level (6). |
| Ethnicity | Ethnicity was determined by the registered country of birth of the participant, and that of the parents (categorical, obtained by the municipal registry). |
| Sex | Sex was obtained by the municipal registry as either male or female (categorical) |
| Hypertension | Blood pressure (BP) was measured twice on the left arm, in a seated position after 5 mins of rest, and hypertension was defined as a mean diastolic BP≥90mmHg and/or systolic BP≥140mmHg and/or use of antihypertensive medication (angiotensin-converting enzyme inhibitors, angiotensin II receptor blockers, diuretics or beta blockers at the time of collection). |
| Smoking status | Smoking status was determined via questionnaire as defined as ‘current smoker’ or ‘non-smoker/former smoker’. |
| Hypercholesterolemia | Total cholesterol was measured in a single measurement of fasting blood samples and hypercholesterolemia was determined by total cholesterol >6.2mmol/l or use of antilipemic medication such as HMG CoA reductase inhibitors, fibrates, bile acid sequestrants, nicotinic acid and derivatives. |
| Diabetes mellitus | Fasting glucose was measured in a single measurement of fasting blood samples and diabetes was determined as fasting glucose ≥7mmol/l, or the use of glucose lowering medication. |
| CVD | CVD was confirmed if participants stated ever having had one or more of the following: Stroke/heart attack/bypass- or stent surgery, estimated via questionnaire (self-reported). |
| Obesity | Obesity was categorised based on the body mass index measured at time of collection and categorised as “normal” <25kg/m^2^; “overweight” 25$\leq$30kg/m^2^; “obese” >30 kg/m^2^. |

*Supplementary table 2: Baseline characteristics across ethnic groups*

|  | | **Women** | | | | | | **Men** | | | | | |
| --- | --- | --- | --- | --- | --- | --- | --- | --- | --- | --- | --- | --- | --- |
|  |  | Dutch | South Asian Surinamese | African Surinamese | Ghanaian | Turkish | Moroccan | Dutch | South Asian Surinamese | African Surinamese | Ghanaian | Turkish | Moroccan |
|  |  | **n=2456** | **n=1663** | **n=2506** | **n=1417** | **n=1961** | **n=2381** | **n=2069** | **n=1363** | **n=1599** | **n=896** | **n=1617** | **n=1502** |
| **Age (years)** | | 45.55 (14.1) | 46.09 (13.1) | 47.81 (12.2) | 43.36 (10.7) | 39.91 (12.19) | 39.46 (12.9) | 46.91 (13.8) | 44.8 (13.6) | 48.11 (12.9) | 46.89 (11.4) | 40.89 (12.1) | 42.07 (12.7) |
| **Educational Level (Missing n=25)** | *Low* | 79 (3.2) | 260 (15.7) | 125 (5.0) | 511 (36.8) | 723 (37.3) | 821 (34.7) | 69 (3.4) | 175 (12.9) | 104 (6.6) | 142 (16.1) | 397 (24.8) | 379 (25.6) |
|  | *Medium-Low* | 361 (14.8) | 563 (34.1) | 814 (32.7) | 504 (36.3) | 387 (20.0) | 365 (15.4) | 279 (13.6) | 440 (32.4) | 644 (40.7) | 403 (45.6) | 491 (30.6) | 324 (21.9) |
|  | *Medium-High* | 504 (20.6) | 462 (27.9) | 907 (36.5) | 310 (22.3) | 555 (28.7) | 788 (33.3) | 483 (23.5) | 417 (30.7) | 539 (34.1) | 260 (29.4) | 458 (28.6) | 495 (33.4) |
|  | *High* | 1497 (61.3) | 368 (22.3) | 641 (25.8) | 63 (4.5) | 272 (14.0) | 392 (16.6) | 1228 (59.6) | 325 (23.9) | 295 (18.6) | 79 (8.9) | 257 (16.0) | 282 (19.1) |
| **Occupational Level** | *Elementary* | 2.0 | 11.4 | 5.0 | 56.6 | 17.3 | 11.5 | 1.3 | 7.2 | 8.6 | 49.0 | 11.9 | 15.1 |
|  | *Low* | 14.8 | 27.8 | 26.8 | 16.2 | 20.4 | 16.3 | 13.7 | 34.3 | 40.3 | 25.1 | 43.3 | 39.0 |
|  | *Intermediate* | 21.9 | 28.4 | 37.4 | 7.0 | 17.2 | 20.7 | 22.1 | 26.3 | 23.6 | 8.2 | 19.7 | 21.0 |
|  | *High or Academic* | 54.6 | 19.0 | 21.5 | 2.6 | 10.6 | 13.1 | 57.6 | 21.9 | 18.1 | 4.4 | 11.8 | 14.1 |
|  | *Not applicable* | 6.7 | 13.4 | 9.4 | 17.7 | 34.4 | 38.5 | 5.2 | 10.3 | 9.4 | 13.4 | 13.3 | 10.9 |
| **Household Composition** | *Living with >1 Adults* | 65.8 | 67.5 | 56.2 | 61.1 | 85.4 | 83.7 | 72.3 | 73.7 | 56.1 | 68.8 | 85.5 | 83.0 |
|  | *Living without adults* | 34.2 | 32.5 | 43.8 | 38.9 | 14.6 | 16.3 | 27.7 | 26.3 | 43.9 | 31.3 | 14.5 | 17.0 |
| **Hypertension (Missing n=9)** | *Yes* | 457 (18.6) | 592 (35.7) | 1135 (45.4) | 697 (49.3) | 41 (21.0) | 370 (15.6) | 669 (32.4) | 560 (4.1) | 743 (46.6) | 520 (58.0) | 480 (29.7) | 342 (22.8) |
| **Smoking Status (Missing n=9)** | *Yes* | 570 (76.7) | 314 (19.0) | 610 (24.4) | 36 (2.6) | 570 (29.2) | 127 (5.3) | 542 (26.2) | 540 (39.7) | 683 (43.0) | 68 (7.6) | 661 (41.2) | 392 (26.3) |
| **Diabetes Mellitus** | *Yes* | 50 (2.0) | 244 (14.7) | 276 (11.0) | 113 (8.0) | 138 (7.0) | 217 (9.1) | 95 (4.6) | 269 (19.7) | 168 (10.5) | 96 (10.7) | 148 (9.2) | 161 (10.7) |
| **Hypercholesterolemia (Missing n=1)** | *Yes* | 533 (21.7) | 471 (28.3) | 491 (19.6) | 223 (15.8) | 323 (16.5) | 260 (10.9) | 492 (23.8) | 463 (34.0) | 305 (19.1) | 190 (21.3) | 319 (19.7) | 195 (13.0) |
| **CVD Prevalence (missing n=5)** | *Yes* | 63 (2.6) | 98 (6.0) | 103 (4.2) | 52 (3.8) | 115 (6.0) | 63 (2.7) | 101 (4.9) | 176 (13.0) | 109 (6.9) | 51 (5.9) | 119 (7.5) | 66 (4.5) |
| **Obesity (Missing n=2)** | *Normal* | 1614 (65.8) | 694 (41.8) | 712 (28.4) | 259 (18.3) | 589 (30.1) | 782 (32.9) | 1104 (53.4) | 620 (45.5) | 664 (41.6) | 314 (35.1) | 404 (25.0) | 527 (35.1) |
|  | *Overweight* | 590 (24.0) | 578 (34.8) | 847 (33.9) | 528 (37.3) | 570 (29.1) | 759 (31.9) | 757 (36.6) | 556 (40.8) | 659 (41.2) | 423 (47.2) | 758 (46.9) | 685 (45.6) |
|  | *Obese* | 250 (10.2) | 390 (23.5) | 941 (37.6) | 628 (44.3) | 801 (40.9) | 838 (35.2) | 208 (10.1) | 186 (13.7) | 275 (17.2) | 158 (17.7) | 454 (28.1) | 289 (19.3) |
| **Time spent on Housework** | *0-3 hours/week* | 409 (16.7) | 220 (13.2) | 331 (13.2) | 297 (21.0) | 340 (17.3) | 324 (13.6) | 651 (31.5) | 448 (32.9) | 426  (26.6) | 414 (46.2) | 1032 (63.8) | 733 (48.8) |
|  | *>3-7.75 hours/week* | 728 (29.6) | 285 (17.0) | 510 (20.4) | 361 (25.5) | 315 (16.1) | 405 (17.0) | 734 (35.5) | 377 (27.7) | 435 (27.2) | 212 (23.7) | 298 (18.4) | 392 (26.1) |
|  | *>7.75-16 hours/week* | 779 (31.7) | 515 (31.0) | 812 (32.4) | 409 (28.9) | 518 (26.4) | 598 (25.1) | 521 (25.2) | 352 (25.8) | 496 (31.0) | 176 (19.6) | 195 (12.1) | 259 (17.2) |
|  | *>16 hours/week* | 540 (22.0) | 643 (38.7) | 853 (34.0) | 350 (24.7) | 788 (40.2) | 1054 (44.3) | 163 (7.9) | 186 (13.6) | 242 (15.1) | 94 (10.5) | 92 (5.7) | 118 (7.9) |
| **Primary earner status (missing n=305)** | *Yes* | 1227 (50.1) | 883 (53.8) | 1810 (73.2) | 915 (66.6) | 530 (27.8) | 734 (31.3) | 1402 (67.9) | 901 (66.4) | 1117 (71.0) | 629 (72.5) | 1156 (72.8) | 1115 (75.0) |
|  | *Equal income* | 491 (20.0) | 254 (15.5) | 236 (9.6) | 186 (13.5) | 344 (18.0) | 301 (12.8) | 441 (21.4) | 190 (14.0) | 184 (11.7) | 121 (13.9) | 152 (9.6) | 142 (9.6) |
|  | *No* | 731 (29.8) | 503 (30.7) | 425 (17.2) | 272 (19.8) | 1034 (54.2) | 1313 (55.9) | 222 (10.8) | 265 (19.5) | 273 (17.3) | 118 (13.6) | 279 (17.6) | 229 (15.4) |
| **Employment Status (missing n=243)** | *Fulltime (>32 hours/week)* | 1064 (43.5) | 605 (3.8) | 1040 (41.8) | 355 (25.8) | 288 (15.0) | 357 (15.2) | 1285 (62.2) | 756 (56.1) | 827 (52.4) | 496 (56.2) | 908 (57.2) | 813 (54.5) |
|  | *12-32 hours/week* | 641 (26.2) | 314 (19.1) | 483 (19.4) | 314 (22.8) | 393 (20.4) | 448 (19.0) | 235 (11.4) | 113 (8.4) | 165 (10.4) | 114 (12.9) | 151 (9.5) | 151 (10.1) |
|  | *<12 hours/week Incl. Homemakers* | 171 (7.0) | 136 (8.3) | 79 (3.2) | 82 (6.0) | 606 (31.5) | 818 (34.8) | 54 (2.6) | 23 (1.7) | 36 (2.3) | 25 (2.8) | 38 (2.4) | 30 (2.0) |
|  | *Not employed* | 572 (23.4) | 591 (35.9) | 884 (35.6) | 626 (45.5) | 637 (33.1) | 729 (31.0) | 492 (23.8) | 455 (33.8) | 551 (34.9) | 248 (28.1) | 490 (30.9) | 498 (33.4) |
| **Female-/Male dominated occupation (missing n=1778) *** | *<25% female workers* | 127 (5.8) | 63 (4.5) | 112 (5.1) | 34 (2.9) | 47 (3.7) | 46 (3.2) | 617 (32.8) | 484 (40.3) | 626 (43.9) | 152 (19.2) | 581 (41.8) | 484 (36.6) |
|  | *26%-50% female* | 706 (32.3) | 271 (16.3) | 293 (34.4) | 186 (16.0) | 277 (21.6) | 228 (16.1) | 742 (39.5) | 4110 (34.2) | 442 (31.0) | 261 (33.0) | 476 (34.2) | 437 (33.1) |
|  | *51-75% female workers* | 804 (36.7) | 664 (47.6) | 762 (34.4) | 810 (69.7) | 648 (50.6) | 644 (45.4) | 418 (22.2) | 254 (21.2) | 281 (19.7) | 366 (46.2) | 305 (21.9) | 348 (26.3) |
|  | *>76% female* | 552 (25.2) | 396 (28.4) | 1048 (47.3) | 132 (11.4) | 309 (24.1) | 502 (35.4) | 103 (5.0) | 52 (4.3) | 78 (5.5) | 13 (1.6) | 29 (2.1) | 53 (4.0) |
|  | *Not applicable* | 117 | 160 | 151 | 169 | 297 | 340 | 79 | 94 | 90 | 54 | 143 | 84 |
| **CKD Prevalence** | *Yes* | 81 (3.3) | 130 (7.8) | 147 (5.9) | 116 (8.2) | 142 (7.2) | 158 (6.6) | 79 (3.8) | 124 (9.1) | 90 (5.6) | 55 (6.1) | 82 (5.1) | 62 (4.1) |
| **CKD (eGFR)** | *Yes* | 29 (1.2) | 35 (2.1) | 28 (1.1) | 16 (1.1) | 10 (0.5) | 13 (0.5) | 35 (1.7) | 34 (2.5) | 27 (1.7) | 15 (1.7) | 11 (0.7) | 9 (0.6) |
| **CKD (ACR)** | *Yes* | 53 (2.2) | 108 (6.5) | 130 (5.2) | 107 (7.6) | 136 (6.9) | 152 (6.4) | 52 (2.5) | 107 (7.9) | 76 (4.8) | 45 (5.0) | 75 (4.6) | 60 (4.0) |
| **CKD (CKD-EPI 2021)** | *Yes* | 70 (2.9) | 120 (7.3) | 162 (6.5) | 123 (8.9) | 137 (7.1) | 155 (6.6) | 66 (3.2) | 116 (8.6) | 105 (6.7) | 70 (7.9) | 78 (4.9) | 61 (4.1) |

*Data presented as means (SDs) and frequencies (percentages); Chronic Kidney Disease (CKD); estimated Glomerular Filtration Rate (eGFR); Cardiovascular Disease (CVD); *not applicable to students, those unable to work and pensioners*

*Supplementary table 3: Associations between gender-related characteristics and CKD in the total population*

|  | Women | | | | | | Men | | | | | | |
| --- | --- | --- | --- | --- | --- | --- | --- | --- | --- | --- | --- | --- | --- |
|  | Model 1 | | Model 2 [Main model] | | Model 3 | | Model 1 | | Model 2 [Main model] | | | Model 3 | |
|  | PR (95%-CI) | p-value | PR (95%-CI) | p-value | PR (95%-CI) | p-value | PR (95%-CI) | p-value | PR (95%-CI) | p-value | P interaction | PR (95%-CI) | p-value |
| **CKD-EPI 2012** | | | | | | | | | | | | | |
| **Time spent on housework (hours/week)** | | | | | | | | | | | | | |
| *0-3* | 1.43 (1.12-1.82) | 0.004 | 1.31 (1.02-1.67) | 0.032 | 1.18 (0.92-1.51) | 0.201 | 1.13 (0.90-1.41) | 0.286 | 1.06 (0.84-1.34) | 0.614 | 0.011 | 1.02 (0.81-1.29) | 0.857 |
| *3-7.75* | Ref |  | Ref |  | Ref |  | Ref |  | Ref |  |  | Ref |  |
| *7.75-16* | 1.07 (0.86-1.33) | 0.540 | 1.05 (0.84-1.30) | 0.685 | 1.04 (0.84-1.30) | 0.719 | 0.87 (0.66-1.13) | 0.280 | 0.87 (0.67-1.14) | 0.308 | 0.514 | 0.88 (0.68-1.15) | 0.352 |
| *>16* | 1.29 (1.05-1.59) | 0.014 | 1.15 (0.93-1.41) | 0.197 | 1.16 (0.94-1.43) | 0.172 | 1.28 (0.96-1.72) | 0.095 | 1.26 (0.94-1.68) | 0.141 | 0.352 | 1.20 (0.89-1.61) | 0.231 |
| *Interaction term** |  |  |  |  |  |  |  |  |  |  | 0.010 |  |  |
| **Primary Earner Status** | | | | | | | | | | | | | |
| *Yes* | Ref |  | Ref |  | Ref |  | Ref |  | Ref |  |  | Ref |  |
| *Equal income* | 0.88 (0.71-1.10) | 0.252 | 0.86 (0.69-1.07) | 0.166 | 0.94 (0.75-1.17) | 0.551 | 0.67 (0.50-0.90) | 0.009 | 0.70 (0.52-0.95) | 0.022 | 0.216 | 0.75 (0.55-1.02) | 0.063 |
| *No* | 1.04 (0.89-1.23) | 0.607 | 0.91 (0.77-1.07) | 0.258 | 0.96 (0.81-1.14) | 0.642 | 1.12 (0.81-1.54) | 0.491 | 1.14 (0.83-1.58) | 0.423 | 0.312 | 1.19 (0.86-1.66) | 0.287 |
| *Interaction term** |  |  |  |  |  |  |  |  |  |  | 0.330 |  |  |
| **Employment Status (hours/week)** | | | | | | | | | | | | | |
| *>32* | Ref |  | Ref |  | Ref |  | Ref |  | Ref |  |  | Ref |  |
| *12-32* | 1.45 (1.15-1.82) | 0.001 | 1.34 (1.06-1.69) | 0.014 | 1.32 (1.05-1.67) | 0.019 | 1.10 (0.79-1.55) | 0.576 | 1.12 (0.79-1.56) | 0.530 | 0.329 | 1.15 (0.82-1.62) | 0.410 |
| *<12* | 1.97 (1.57-2.48) | <0.001 | 1.44 (1.12-1.86) | 0.005 | 1.44 (1.11-1.86) | 0.006 | 0.49 (0.18-1.32) | 0.157 | 0.49 (0.18-1.33) | 0.161 | 0.038 | 0.61 (0.23-1.64) | 0.324 |
| *Not employed* | 1.88 (1.55-2.29) | <0.001 | 1.61 (1.31-1.98) | <0.001 | 1.37 (1.12-1.71) | 0.002 | 1.50 (1.23-1.83) | <0.001 | 1.43 (1.17-1.75) | <0.001 | 0.596 | 1.33 (1.08-1.63) | 0.007 |
| *Interaction term** |  |  |  |  |  |  |  |  |  |  | 0.088 |  |  |
| **Female-/Male dominated Occupation (% of female workers)** | | | | | | | | | | | | | |
| *<25%* | Ref |  | Ref |  | Ref |  | Ref |  | Ref |  |  | Ref |  |
| *26%-50%* | 2.04 (0.47-8.75) | 0.339 | 2.1 (0.49-9.01) | 0.320 | 1.05 (0.86-1.30) | 0.623 | 0.91 (0.58-1.43) | 0.674 | 0.99 (0.63-1.57) | 0.979 | 0.337 | 1.28 (0.72-2.29) | 0.407 |
| *51%-75%* | 2.29 (0.56-9.41) | 0.250 | 2.21 (0.54-9.09) | 0.272 | 0.97 (0.74-1.3) | 0.792 | 1.13 (0.72-1.77) | 0.601 | 1.27 (0.81-2.01) | 0.302 | 0.701 | 1.40 (0.79-2.48) | 0.249 |
| *>76%* | 1.18 (0.28-5.05) | 0.819 | 1.26 (0.29-5.35) | 0.759 | 1.07 (0.69-1.65) | 0.776 | 1.05 (0.45-2.46) | 0.916 | 1.29 (0.39-3.10) | 0.565 | 0.768 | 1.28 (0.71-2.25) | 0.421 |
| *Interaction term** |  |  |  |  |  |  |  |  |  |  | 0.428 |  |  |

*PR=Prevalence Ratio; Ref =Reference category; CI =confidence interval); P interaction sex*gender-related characteristic only calculated for main model (model 2) across all categories individually and for the overall variable; *refers to the overall interaction term of the variable; Model 1: Age-adjusted; Model 2: Adjusts for age, education, and ethnicity; Model 3: Adjusts for Age, education, ethnicity, hypertension, diabetes, hypercholesterolemia, obesity, smoking status, and CVD;  Significant results (p-values <0.05);*

*Supplementary table 4: Associations between gender-related characteristics and eGFR <60 ml/min/1.73m^2^ in the total population*

|  | Women | | | | | | Men | | | | | |
| --- | --- | --- | --- | --- | --- | --- | --- | --- | --- | --- | --- | --- |
|  | Model 1 | | Model 2 [Main model] | | Model 3 | | Model 1 | | Model 2 [Main model] | | Model 3 | |
|  | PR (95%-CI) | p-value | PR (95%-CI) | p-value | PR (95%-CI) | p-value | PR (95%-CI) | p-value | PR (95%-CI) | p-value | PR (95%-CI) | p-value |
| **eGFR <60 ml/min/1.73m^2^** | | | | | | | | | | | | |
| **Time spent on housework (hours/week)** | | | | | | | | | | | | |
| *0-3* | 1.19 (0.66-2.14) | 0.572 | 1.09 (0.51-2.34) | 0.825 | 0.93 (0.53-1.78) | 0.927 | 0.91 (0.59-1.39) | 0.653 | 0.91 (0.59-1.40) | 0.664 | 0.84 (0.54-1.3) | 0.428 |
| *3-7.75* | Ref |  | Ref |  | Ref |  | Ref |  | Ref |  | Ref |  |
| *7.75-16* | 0.98 (0.59-1.62) | 0.934 | 0.98 (0.59-1.61) | 0.922 | 0.97 (0.59-1.60) | 0.909 | 0.72 (0.44-1.16) | 0.160 | 0.706 (0.434-1.148) | 0.165 | 0.69 (0.42-1.12) | 0.136 |
| *>16* | 0.88 (0.54-1.43) | 0.602 | 0.88 (0.54-1.43) | 0.603 | 0.88 (0.54-1.44) | 0.620 | 0.91 (0.52-1.59) | 0.636 | 0.873 (0.499-1.530) | 0.655 | 0.87 (0.49-1.52) | 0.613 |
| **Primary Earner Status** | | | | | | | | | | | | |
| *Yes* | Ref |  | Ref |  | Ref |  | Ref |  | Ref |  | Ref |  |
| *Equal income* | 1.10 (0.70-1.72) | 0.693 | 1.09 (0.69-1.71) | 0.714 | 1.18 (0.75-1.86) | 0.479 | 0.63 (0.35-1.14) | 0.127 | 0.640 (0.352-1.164) | 0.144 | 0.70 (0.38-1.27) | 0.241 |
| *No* | 0.61 (0.38-0.98) | 0.039 | 0.58 (0.36-0.93) | 0.023 | 0.62 (0.39-1.01) | 0.052 | 1.15 (0.58-2.28) | 0.681 | 1.15 (0.58-2.27) | 0.697 | 1.23 (0.62-2.44) | 0.560 |
| **Employment Status (hours/week)** | | | | | | | | | | | | |
| *>32* | Ref |  | Ref |  | Ref |  | Ref |  | Ref |  | Ref |  |
| *12-32* | 1.21 (0.64-2.32) | 0.557 | 1.16 (0.61-2.23) | 0.645 | 1.14 (0.60-2.18) | 0.692 | 1.15 (0.55-2.37) | 0.712 | 1.15 (0.55-2.37) | 0.714 | 1.14 (0.55-2.38) | 0.718 |
| *<12* | 1.31 (0.70-2.47) | 0.405 | 1.11 (0.56-2.22) | 0.766 | 1.18 (0.59-2.38) | 0.636 | 0.47 (0.07-3.47) | 0.463 | 0.47 (0.06-3.42) | 0.454 | 0.56 (0.08-4.08) | 0.565 |
| *Not employed* | 1.58 (0.93-2.66) | 0.089 | 1.41 (0.82-2.43) | 0.210 | 1.25 (0.72-2.16) | 0.423 | 1.62 (1.06-2.47) | 0.027 | 1.57 (1.02-2.42) | 0.040 | 1.45 (0.94-2.25) | 0.094 |
| **Female-/Male dominated Occupation (% of female workers)** | | | | | | | | | | | | |
| *<25%* | Ref |  | Ref |  | Ref |  | Ref |  | Ref |  | Ref |  |
| *26%-50%* | 2.04 (0.47-8.75) | 0.339 | 2.10 (0.49-9.01) | 0.320 | 2.20 (0.51-9.49) | 0.291 | 0.91 (0.58-1.43) | 0.674 | 0.99 (0.63-1.57) | 0.979 | 0.96 (0.61-1.52) | 0.865 |
| *51%-75%* | 2.29 (0.56-9.41) | 0.250 | 2.21 (0.54-9.09) | 0.272 | 2.33 (0.56-9.62) | 0.244 | 1.13 (0.72-1.77) | 0.601 | 1.27 (0.81-2.01) | 0.302 | 1.24 (0.78-1.96) | 0.363 |
| *>76%* | 1.18 (0.28-5.05) | 0.819 | 1.26 (0.29-5.35) | 0.759 | 1.29 (0.30-5.55) | 0.728 | 1.05 (0.45-2.46) | 0.916 | 1.29 (0.39-3.10) | 0.565 | 0.35 (0.56-3.25) | 0.507 |

*PR=Prevalence Ratio; Ref =Reference category; CI =confidence interval); P interaction sex*gender-related characteristic only calculated for main model (model 2) across all categories individually and for the overall variable; *refers to the overall interaction term of the variable; Model 1: Age-adjusted; Model 2: Adjusts for age, education, and ethnicity; Model 3: Adjusts for Age, education, ethnicity, hypertension, diabetes, hypercholesterolemia, obesity, smoking status, and CVD;  Significant results (p-values <0.05);*

*Supplementary table 5: Associations between gender-related characteristics and ACR ≥3mg/mml in the total population*

|  | Women | | | | | | Men | | | | | |
| --- | --- | --- | --- | --- | --- | --- | --- | --- | --- | --- | --- | --- |
|  | Model 1 | | Model 2 [Main model] | | Model 3 | | Model 1 | | Model 2 [Main model] | | Model 3 | |
|  | PR (95%-CI) | p-value | PR (95%-CI) | p-value | PR (95%-CI) | p-value | PR (95%-CI) | p-value | PR (95%-CI) | p-value | PR (95%-CI) | p-value |
| **ACR ≥3mg/mml** | | | | | | | | | | | | |
| **Time spent on housework (hours/week)** | | | | | | | | | | | | |
| *0-3* | 1.41 (1.09-1.81) | 0.008 | 1.27 (0.98-1.64) | 0.067 | 1.14 (0.88-1.48) | 0.317 | 1.24 (0.97-1.59) | 0.083 | 1.15 (0.89-1.48) | 0.290 | 1.11 (0.86-1.43) | 0.437 |
| *3-7.75* | Ref |  | Ref |  | Ref |  | Ref |  | Ref |  | Ref |  |
| *7.75-16* | 1.03 (0.82-1.30) | 0.779 | 1.00 (0.80-1.27) | 0.983 | 1.00 (0.79-1.26) | 0.975 | 0.93 (0.70-1.25) | 0.641 | 0.95 (0.71-1.27) | 0.711 | 0.97 (0.72-1.3) | 0.821 |
| *>16* | 1.32 (1.06-1.64) | 0.012 | 1.14 (0.92-1.42) | 0.246 | 1.15 (0.92-1.43) | 0.218 | 1.39 (1.0-1.92) | 0.048 | 1.36 (0.99-1.88) | 0.062 | 1.29 (0.93-1.79) | 0.128 |
| **Primary Earner Status** | | | | | | | | | | | | |
| *Yes* | Ref |  | Ref |  | Ref |  | Ref |  | Ref |  | Ref |  |
| *Equal income* | 0.83 (0.66-1.06) | 0.133 | 0.80 (0.63-1.02) | 0.070 | 0.88 (0.69-1.13) | 0.310 | 0.69 (0.50-0.96) | 0.026 | 0.74 (0.54-1.02) | 0.065 | 0.79 (0.57-1.1) | 0.157 |
| *No* | 1.07 (0.90-1.26) | 0.467 | 0.90 (0.75-1.07) | 0.228 | 0.95 (0.79-1.14) | 0.594 | 1.03 (0.72-1.46) | 0.883 | 1.06 (0.74-1.51) | 0.757 | 1.1 (0.77-1.58) | 0.590 |
| **Employment Status (hours/week)** | | | | | | | | | | | | |
| *>32* | Ref |  | Ref |  | Ref |  | Ref |  | Ref |  | Ref |  |
| *12-32* | 1.45 (1.14-1.85) | 0.002 | 1.30 (1.02-1.66) | 0.035 | 1.28 (1.00-1.64) | 0.048 | 1.10 (0.77-1.58) | 0.606 | 1.12 (0.78-1.60) | 0.552 | 1.16 (0.81-1.67) | 0.421 |
| *<12* | 2.03 (1.60-2.58) | <0.001 | 1.37 (1.05-1.78) | 0.021 | 1.35 (1.03-1.77) | 0.031 | 0.43 (0.14-1.34) | 0.143 | 0.43 (0.14-1.34) | 0.147 | 0.54 (0.17-1.69) | 0.288 |
| *Not employed* | 1.80 (1.46-2.22) | <0.001 | 1.47 (1.18-1.83) | <0.001 | 1.25 (1.0-1.57) | 0.050 | 1.49 (1.20-1.84) | <0.001 | 1.40 (1.13-1.74) | 0.002 | 1.30 (0.17-1.69) | 0.019 |
| **Female-/Male dominated Occupation (% of female workers)** | | | | | | | | | | | | |
| *<25%* | Ref |  | Ref |  | Ref |  | Ref |  | Ref |  | Ref |  |
| *26%-50%* | 1.12 (0.91-1.38) | 0.281 | 0.95 (0.77-1.18) | 0.639 | 0.97 (0.78-1.21) | 0.802 | 1.43 (0.76-2.67) | 0.264 | 1.34 (0.69-2.58) | 0.389 | 1.35 (0.7-2.62) | 0.370 |
| *51%-75%* | 0.84 (0.64-1.11) | 0.228 | 0.85 (0.65-1.12) | 0.253 | 0.90 (0.68-1.19) | 0.446 | 1.64 (0.89-3.04) | 0.113 | 1.58 (0.82-3.01) | 0.169 | 1.55 (0.81-2.97) | 0.185 |
| *>76%* | 1.02 (0.64-1.61) | 0.947 | 1.01 (0.64-1.61) | 0.952 | 1.08 (0.68-1.71) | 0.753 | 1.41 (0.76-2.61) | 0.273 | 1.31 (0.69-2.52) | 0.411 | 1.33 (0.69-2.55) | 0.398 |

*PR=Prevalence Ratio; Ref =Reference category; CI =confidence interval); P interaction sex*gender-related characteristic only calculated for main model (model 2) across all categories individually and for the overall variable; *refers to the overall interaction term of the variable; Model 1: Age-adjusted; Model 2: Adjusts for age, education, and ethnicity; Model 3: Adjusts for Age, education, ethnicity, hypertension, diabetes, hypercholesterolemia, obesity, smoking status, and CVD;  Significant results (p-values <0.05);*

*Supplementary table 6: Additional sensitivity analyses in the total population*

|  | | **Women** | | **Men** | |
| --- | --- | --- | --- | --- | --- |
|  | Model 2 | PR | p-value | PR | p-value |
| **CKD (CKD-EPI 2012)^▪^** | | | | | |
| Time spent on housework (restricted to those living with another adult(s)) | *0-3 hours/week* | 1.23 (0.91-1.66) | 0.185 | 1.05 (0.80-1.38) | 0.731 |
|  | *3-7.75 hours/week* | Ref |  | Ref |  |
|  | *7.75-16 hours/week* | 1.02 (0.78-1.33) | 0.875 | 0.75 (0.53-1.06) | 0.097 |
|  | *>16 hours/week* | 1.13 (0.88-1.45) | 0.352 | 1.13 (0.77-1.67) | 0.535 |
| Primary Earner Status (Restricted to those living with another adult(s)) | *Yes* | Ref |  | Ref |  |
|  | *No, equal income* | 0.80 (0.62-1.01) | 0.065 | 0.66 (0.47-0.91) | 0.013 |
|  | *No* | 0.78 (0.64-0.95) | 0.015 | 1.10 (0.77-1.56) | 0.610 |
| Employment Status (Excluding students, pensioners, and those unable to work) | *>32 hours/week* | Ref |  | Ref |  |
|  | *12-32hours/week* | 1.33 (1.05-1.68) | 0.018 | 1.12 (0.80-1.57) | 0.511 |
|  | *<12 hours/week incl. Homemakers* | 1.43 (1.10-1.86) | 0.008 | 0.50 (0.19-1.35) | 0.170 |
|  | *Not employed* | 1.43 (1.11-1.84) | 0.006 | 1.26 (0.96-1.64) | 0.098 |
| Female-/Male dominated Occupation (Excluding students, pensioners, and those unable to work) | *<25% female workers* | Ref |  | Ref |  |
|  | *26-50% female workers* | 0.97 (0.77-1.22) | 0.779 | 1.18 (0.58-2.37) | 0.649 |
|  | *51-75% female workers* | 0.81 (0.60-1.09) | 0.158 | 1.40 (0.71-2.77) | 0.337 |
|  | *>76% female workers* | 0.95 (0.57-1.58) | 0.847 | 1.29 (0.65-2.56) | 0.464 |
| Female-/Male dominated Occupation - Model 2 + Employment Status & Occupational level | *<25% female workers* | Ref |  | Ref |  |
|  | *26-50% female workers* | 0.94 (0.59-1.49) | 0.783 | 1.13 (0.90-1.42) | 0.287 |
|  | *51-75% female workers* | 0.95 (0.61-1.47) | 0.808 | 0.96 (0.74-1.23) | 0.730 |
|  | *>76% female workers* | 1.01 (0.64-1.58) | 0.970 | 0.86 (0.49-1.54) | 0.617 |
| **CKD-EPI 2021*** | | | | | |
| Time spent on housework | *0-3 hours/week* | 1.32 (1.04-1.69) | 0.024 | 1.12 (0.89-1.41) | 0.325 |
|  | *3-7.75 hours/week* | Ref |  | Ref |  |
|  | *7.75-16 hours/week* | 1.04 (0.83-1.29) | 0.741 | 0.93 (0.72-1.22) | 0.611 |
|  | *>16 hours/week* | 1.15 (0.93-1.41) | 0.199 | 1.32 (0.99-1.77) | 0.061 |
| Primary Earner Status | *Yes* | Ref |  | Ref |  |
|  | *No, equal income* | 0.83 (0.67-1.04) | 0.100 | 0.71 (0.53-0.96) | 0.026 |
|  | *No* | 0.87 (0.73-1.02) | 0.089 | 1.16 (0.84-1.59) | 0.380 |
| Employment Status | *>32 hours/week* | Ref |  | Ref |  |
|  | *12-32hours/week* | 1.36 (1.08-1.72) | 0.010 | 1.13 (0.81-1.58) | 0.458 |
|  | *<12 hours/week incl. Homemakers* | 1.40 (1.08-1.81) | 0.010 | 0.36 (0.11-1.12) | 0.077 |
|  | *Not employed* | 1.63 (1.33-2.01) | 0.010 | 1.37 (1.12-1.67) | 0.002 |
| Female-/Male dominated Occupation | *<25% female workers* | Ref |  | Ref |  |
|  | *26-50% female workers* | 0.96 (0.78-1.17) | 0.659 | 1.37 (0.75-2.51) | 0.302 |
|  | *51-75% female workers* | 0.85 (0.66-1.10) | 0.219 | 1.64 (0.91-2.96) | 0.101 |
|  | *>76% female workers* | 0.96 (0.62-1.48) | 0.836 | 1.40 (0.78-2.54) | 0.262 |

**^▪^***Main model analyses with sample restriction methods defined in the sample;* **Main model analyses conducted using the CKD-EPI 2021 equation rather than the CKD-EPI2012 equation; Model 2: Adjusted for Age, Ethnicity and Educational level; PR = Prevalence Ratios, Significant associations p-value <0.05; 95% Confidence Intervals; Ref= Reference category*

*Supplementary table 7: Associations between gender-related characteristics and CKD in women across ethnic groups*

| **Women** | Dutch | | South-Asian Surinamese | | African Surinamese | | Ghanaian | | Turkish | | Moroccan | |
| --- | --- | --- | --- | --- | --- | --- | --- | --- | --- | --- | --- | --- |
|  | PR | p-value | PR | p-value | PR | p-value | PR | p-value | PR | p-value | PR | p-value |
| **CKD (CKD-EPI 2012)** | | | | | | | | | | | | |
| Time spent on housework | | | | | | | | | | | | |
| *0-3 hours/week* | 3.61 (1.67-7.86) | 0.001 | 0.50 (0.24-1.03) | 0.061 | 1.4 (0.81-2.44) | 0.231 | 1.36 (0.79-2.34) | 0.271 | 1.21 (0.71-2.06) | 0.487 | 1.15 | 0.660 |
| *3-7.75 hours/week* | Ref |  | Ref |  | Ref |  | Ref |  | Ref |  | Ref |  |
| *7.75-16 hours/week* | 2.25 (1.09-4.66) | 0.029 | 0.59 (0.36-0.99) | 0.045 | 0.94 (0.58-1.53) | 0.806 | 1.14 (0.67-1.92) | 0.629 | 0.72 (0.42-1.24) | 0.236 | 1.395 | 0.220 |
| *>16 hours/week* | 2.50 (1.17-5.37) | 0.018 | 0.78 (0.50-1.24) | 0.297 | 1.07 (0.67-1.71) | 0.771 | 1.26 (0.74-2.15) | 0.388 | 0.81 (0.50-1.31) | 0.395 | 1.260 | 0.362 |
| **Primary Earner Status** | | | | | | | | | | | | |
| *Yes* | Ref |  | Ref |  | Ref |  | Ref |  | Ref |  | Ref |  |
| *No, equal income* | 0.60 (0.31-1.15) | 0.123 | 0.94 (0.57-1.56) | 0.812 | 0.89 (0.49-1.63) | 0.714 | 0.97 (0.57-1.75) | 0.910 | 0.75 (0.43-1.29) | 0.297 | 1.15 (0.70-1.89) | 0.585 |
| *No* | 0.73 (0.44-1.21) | 0.218 | 0.99 (0.64-1.52) | 0.952 | 1.17 (0.74-1.87) | 0.506 | 0.56 (0.30-1.03) | 0.061 | 1.03 (0.69-1.53) | 0.890 | 1.09 (0.6-1.57) | 0.643 |
| **Employment Status** | | | | | | | | | | | | |
| *>32 hours/week* | Ref |  | Ref |  | Ref |  | Ref |  | Ref |  | Ref |  |
| *12-32hours/week* | 0.96 (0.51-1.81) | 0.898 | 1.52 (0.87-2.66) | 0.141 | 1.57 (0.97-2.55) | 0.067 | 1.15 (0.67-1.98) | 0.620 | 1.64 (0.78-3.46) | 0.193 | 1.24 (0.66-2.32) | 0.503 |
| *<12 hours/week incl. Homemakers* | 1.48 (0.64-3.43) | 0.365 | 1.81 (0.94-3.50) | 0.078 | 2.78 (1.34-5.78) | 0.006 | 0.84 (0.32-2.19) | 0.715 | 2.12 (1.04-4.33) | 0.039 | 1.23 (0.67-2.26) | 0.503 |
| *Not employed* | 1.78 (1.00-3.15) | 0.050 | 1.79 (1.12-2.87) | 0.015 | 1.68 (1.11-2.54) | 0.014 | 1.17 (0.73-1.87) | 0.506 | 2.09 (1.04-04.19) | 0.038 | 1.24 (0.68-2.25) | 0.485 |
| **Female-/Male dominated Occupation** | | | | | | | | | | | | |
| *<25% female workers* | Ref |  | Ref |  | Ref |  | Ref |  | Ref |  | Ref |  |
| *26-50% female workers* | 0.86 (0.29-2.55) | 0.789 | 2.11 (0.49-9.09) | 0.314 | 1.60 (0.45-5.69) | 0.465 | 0.61 (0.19-1.90) | 0.392 | 0.56 (0.21-1.52) | 0.256 | 0.51 (0.18-1.42) | 0.197 |
| *51-75% female workers* | 1.23 (0.44-3.48) | 0.696 | 2.10 (0.51-8.63) | 0.305 | 2.44 (0.76-7.84) | 0.133 | 0.75 (0.27-2.05) | 0.568 | 0.40 (0.16-1.04) | 0.061 | 0.43 (0.17-1.11) | 0.080 |
| *>76% Female workers* | 0.94 (0.32-2.78) | 0.909 | 2.56 (0.62-10.67) | 0.196 | 1.85 (0.58-5.91) | 0.302 | 0.52 (0.15-1.87) | 0.317 | 0.66 (0.25-1.77) | 0.409 | 0.39 (0.15-1.02) | 0.055 |

*PR=Prevalence Ratio; Ref =Reference category; CI =confidence interval; Model 2: Adjusts for age, education, and ethnicity; . groups too small to calculate estimate*

*Supplementary table 8: Associations between gender-related characteristics and eGFR<60ml/min/1.73m^2^ in women across ethnic groups*

| **Women** | Dutch | | South-Asian Surinamese | | African Surinamese | | Ghanaian | | Turkish | | Moroccan | |
| --- | --- | --- | --- | --- | --- | --- | --- | --- | --- | --- | --- | --- |
|  | PR | p-value | PR | p-value | PR | p-value | PR | p-value | PR | p-value | PR | p-value |
| **eGFR<60ml/min/1.73m^2^** | | | | | | | | | | | | |
| **Time spent on housework** | | | | | | | | | | | | |
| *0-3 hours/week* | 2.36 (0.48-11.68) | 0.294 | 0.54 (0.16-1.74) | 0.300 | 1.44 (0.36-5.76) | 0.609 | 1.70 (0.38-7.62) | 0.487 | 0.47 (0.09-2.55) | 0.378 | 2.10 (0.35-12.55) | 0.418 |
| *3-7.75 hours/week* | Ref |  | Ref |  | Ref |  | Ref |  | Ref |  | Ref |  |
| *7.75-16 hours/week* | 3.27 (0.94-11.35) | 0.063 | 0.50 (0.20-1.23) | 0.133 | 1.02 (0.30-3.47) | 0.979 | 2.03 (0.50-8.14) | 0.320 | 0.19 (0.2-1.79) | 0.148 | 0.37 (0.3-4.14) | 0.422 |
| *>16 hours/week* | 1.93 (0.49-7.58) | 0.344 | 0.43 (0.18-1.02) | 0.054 | 1.65 (0.54-5.07) | 0.379 | 1.39 (0.28-6.99) | 0.690 | 0.35 (0.08-2.55) | 0.182 | 1.33 (0.27-6.53) | 0.727 |
| **Primary Earner Status** | | | | | | | | | | | | |
| *Yes* | Ref |  | Ref |  | Ref |  | Ref |  | Ref |  | Ref |  |
| *No, equal income* | 1.09 (0.45-2.62) | 0.852 | 2.52 (1.12-5.64) | 0.025 | 1.03 (0.31-3.44) | 0.957 | 1.00 (0.23-4.44) | 1.000 | 0.59 (0.12-2.92) | 0.517 | 0.28 (0.3-2.24) | 0.229 |
| *No* | 0.46 (0.17-1.26) | 0.131 | 1.73 (0.75-4.01) | 0.199 | 0.70 (0.17-2.99) | 0.634 | 0.54 (0.7-4.16) | 0.552 | 0.27 (0.05-1.34) | 0.109 | 0.39 (0.11-1.32) | 0.130 |
| **Employment Status** | | | | | | | | | | | | |
| *>32 hours/week* | Ref |  | Ref |  | Ref |  | Ref |  | Ref |  | Ref |  |
| *12-32hours/week* | 0.74 (0.24-2.28) | 0.597 | 1.08 (0.24-4.84) | 0.924 | 0.31 (0.04-2.59) | 0.280 | 2.40 (0.40-14.48) | 0.340 | <0.001 | . | . | . |
| *<12 hours/week incl. Homemakers* | 0.62 (0.12-3.20) | 0.566 | 1.77 (0.44-7.17) | 0.423 | 1.23 (0.14-10.51) | 0.851 | <0.00 (0.0-0.0) | . | . | . | . | . |
| *Not employed* | 0.96 (0.38-2.74) | 0.939 | 1.08 (0.40-4.30) | 0.659 | 1.70 (0.62-4.65) | 0.301 | 2.69 (0.59-12.26) | 0.201 | . | . | . | . |
| **Female-/Male dominated Occupation** | | | | | | | | | | | | |
| *<25% female workers* | Ref |  | Ref |  | Ref |  | Ref |  | Ref |  | Ref |  |
| *26-50% female workers* | 1.71 (0.22-13.57) | 0.610 | . | . | . | . | <0.00 (0.0-0.0) | 1.000 | . | . | 0.253 (0.0-0.0) | 1.000 |
| *51-75% female workers* | 1.24 (0.16-9.69) | 0.836 | . | . | . | . | 0.48 (0.6-3.77) | 0.488 | . | . | . | . |
| *>76% Female workers* | 0.78 (0.9-6.69) | 0.817 | . | . | . | . | <0.00 (0.0-0.0) | . | . | . | . | . |

*PR=Prevalence Ratio; Ref =Reference category; CI =confidence interval; Model 2: Adjusts for age, education, and ethnicity; . groups too small to calculate estimate*

*Supplementary table 9: Associations between gender-related characteristics and ACR ≥3mg/mml in women across ethnic groups*

| **Women** | Dutch | | South-Asian Surinamese | | African Surinamese | | Ghanaian | | Turkish | | Moroccan | |
| --- | --- | --- | --- | --- | --- | --- | --- | --- | --- | --- | --- | --- |
|  | PR | p-value | PR | p-value | PR | p-value | PR | p-value | PR | p-value | PR | p-value |
| **ACR ≥3mg/mml** | | | | | | | | | | | | |
| **Time spent on housework** | | | | | | | | | | | | |
| *0-3 hours/week* | 3.85 (1.56-9.52) | 0.004 | 0.51 (0.24-1.10) | 0.087 | 1.45 (0.82-2.55) | 0.202 | 1.35 (0.76-2.37) | 0.305 | 1.18 (0.68-2.04) | 0.555 | 1.01 (0.51-1.98) | 0.982 |
| *3-7.75 hours/week* | Ref |  | Ref |  | Ref |  | Ref |  | Ref |  | Ref |  |
| *7.75-16 hours/week* | 1.65 (0.65-4.15) | 0.292 | 0.55 (0.31-0.97) | 0.038 | 0.91(0.55-1.51) | 0.705 | 1.16 (0.68-2.00) | 0.585 | 0.73 (0.42-1.27) | 0.268 | 1.48 (0.86-2.54) | 0.157 |
| *>16 hours/week* | 3.0 (1.21-7.43) | 0.018 | 0.83 (0.51-1.37) | 0.466 | 0.97 (0.59-1.60) | 0.915 | 1.23 0.71-2.15) | 0.464 | 0.83 (0.50-1.35) | 0.445 | 1.31 (0.79-2.18) | 0.299 |
| **Primary Earner Status** | | | | | | | | | | | | |
| *Yes* | Ref |  | Ref |  | Ref |  | Ref |  | Ref |  | Ref |  |
| *No, equal income* | 0.42 (0.16-1.07) | 0.068 | 0.78 (0.44-1.40) | 0.409 | 0.86 (0.45-1.64) | 0.637 | 0.90 (0.51-1.58) | 0.715 | 0.76 (0.43-1.34) | 0.344 | 1.17 (0.70-1.96) | 0.539 |
| *No* | 0.86 (0.47-1.56) | 0.610 | 0.84 (0.52-1.35) | 0.841 | 1.19 (0.73-1.93) | 0.489 | 0.56 (0.30-1.04) | 0.064 | 1.06 (0.71-1.60) | 0.769 | 1.12 (0.77-1.62) | 0.558 |
| **Employment Status** | | | | | | | | | | | | |
| *>32 hours/week* | Ref |  | Ref |  | Ref |  | Ref |  | Ref |  | Ref |  |
| *12-32hours/week* | 1.05 (0.49-2.25) | 0.906 | 1.45 (0.81-2.59) | 0.208 | 1.64 (1.00-2.70) | 0.050 | 1.12 (0.64-1.96) | 0.687 | 1.50 (0.71-3.21) | 0.290 | 1.19 (0.63-2.24) | 0.594 |
| *<12 hours/week incl. Homemakers* | 1.92 (0.72-5.14) | 0.195 | 1.41 (0.67-2.95) | 0.369 | 2.73 (1.26-5.91) | 0.011 | 0.84 (0.32-2.19) | 0.714 | 2.07 (1.01-4.25) | 0.047 | 1.22 (0.66-2.24) | 0.526 |
| *Not employed* | 1.96 (0.98-1.02) | 0.058 | 1.58 (0.96-2.59) | 0.071 | 1.55 (1.00-2.40) | 0.050 | 1.06 (0.65-1.72) | 0.820 | 2.10 (1.04-4.21) | 0.037 | 1.17 (0.64-2.13) | 0.609 |
| **Female-/Male dominated Occupation** | | | | | | | | | | | | |
| *<25% female workers* | Ref |  | Ref |  | Ref |  | Ref |  | Ref |  | Ref |  |
| *26-50% female workers* | 0.56 (0.15-2.06) | 0.773 | 1.66 (0.38-7.28) | 0.114 | 1.47 (0.41-5.27) | 0.225 | 0.81 (0.23-2.88) | 0.466 | 0.57 (0.21-1.53) | 0.118 | 0.51 (0.18-1.43) | 0.760 |
| *51-75% female workers* | 1.17 (0.35-3.89) | 0.140 | 1.68 (0.41-6.97) | 0.213 | 2.12 (0.66-6.84) | 0.739 | 0.89 (0.28-2.85) | 0.676 | 0.38 (0.15-0.99) | 0.809 | 0.42 (0.16-1.09) | 0.429 |
| *>76% Female workers* | 1.06 (0.30-3.68) | 0.932 | 2.46 (0.59-10.28) | 0.216 | 1.64 (0.51-5.28) | 0.406 | 0.66 (0.16-2.66) | 0.555 | 0.62 (0.23-1.66) | 0.338 | 0.38 (0.15-1.02) | 0.055 |

*PR=Prevalence Ratio; Ref =Reference category; CI =confidence interval; Model 2: Adjusts for age, education, and ethnicity; . groups too small to calculate estimate*

*Supplementary table 10: Associations between gender-related characteristics and CKD in men across ethnic groups*

|  | Dutch | | South-Asian Surinamese | | African Surinamese | | Ghanaian | | Turkish | | Moroccan | |
| --- | --- | --- | --- | --- | --- | --- | --- | --- | --- | --- | --- | --- |
|  | PR | p-value | PR | p-value | PR | p-value | PR | p-value | PR | p-value | PR | p-value |
| **CKD (CKD-EPI 2012)** | | | | | | | | | | | | |
| **Time spent on housework** | | | | | | | | | | | | |
| *0-3 hours/week* | 1.06 (0.62-1.83) | 0.830 | 1.08 (0.66-1.75) | 0.767 | 0.71 (0.40-1.25) | 0.23 | 1.10 (0.56-2.18) | 0.79 | 1.19 (0.64-2.19) | 0.588 | 1.28 (0.69-2.39) | 0.439 |
| *3-7.75 hours/week* | Ref |  | Ref |  | Ref |  | Ref |  | Ref |  | Ref |  |
| *7.75-16 hours/week* | 0.77 (0.42-1.40) | 0.390 | 0.90 (0.54-1.52) | 0.696 | 0.72 (0.43-1.22) | 0.224 | 1.21 (0.54-2.70) | 0.643 | 1.05 (0.45-2.46) | 0.907 | 0.53 (0.19-1.47) | 0.223 |
| *>16 hours/week* | 0.897 (0.41-1.99) | 0.790 | 1.44 (0.85-2.43) | 0.172 | 0.69 (0.37-1.32) | 0.263 | 0.98 (0.34-2.79) | 0.968 | 1.49 (0.59-3.74) | 0.398 | 1.98 (0.86-4.58) | 0.111 |
| **Primary Earner Status** | | | | | | | | | | | | |
| *Yes* | Ref |  | Ref |  | Ref |  | Ref |  | Ref |  | Ref |  |
| *No, equal income* | 0.77 (0.40-1.47) | 0.428 | 0.47 (0.24-9.4) | 0.032 | 0.63 (0.29-1.38) | 0.249 | 1.60 (0.81-3.16) | 0.175 | 0.93 (0.44-1.93) | 0.835 | 0.34 (0.08-1.38) | 0.129 |
| *No* | 0.87 (0.35-2.18) | 0.766 | 0.74 (0.340-1.62) | 0.455 | 1.08 (0.55-2.1) | 0.825 | 1.92 (0.76-4.87) | 0.169 | 1.08 (0.43-2.72) | 0.871 | 1.92 (0.90-4.13) | 0.093 |
| **Employment Status** | | | | | | | | | | | | |
| *>32 hours/week* | Ref |  | Ref |  | Ref |  | Ref |  | Ref |  | Ref |  |
| *12-32 hours/week* | 0.86 (0.36-2.07) | 0.734 | 1.28 (0.62-2.61) | 0.507 | 1.01 (0.45-2.27) | 0.975 | 1.39 (0.60-3.23) | 0.439 | 1.48 (0.69-3.18) | 0.311 | 0.68 (0.21-2.26) | 0.533 |
| *<12 hours/week incl. Homemakers* | 0.93 (0.22-3.94) | 0.918 | <0.00 (0.0-0.0) | . | 0.69 (0.10-5.01) | 0.712 | 0.92 (0.13-6.80) | 0.936 | <0.00 (0.0-0.0) | . | <0.00 (0.0-0.0) | . |
| *Not employed* | 1.45 (0.83-2.54) | 0.192 | 1.37(0.91-2.08) | 0.135 | 1.19 (0.75-1.89) | 0.470 | 1.59 (0.89-2.86) | 0.119 | 1.29 (0.80-2.10) | 0.297 | 1.57 (0.91-2.70) | 0.105 |
| **Female-/Male dominated Occupation** | | | | | | | | | | | | |
| *<25% female workers* | Ref |  | Ref |  | Ref |  | Ref |  | Ref |  | Ref |  |
| *26-50% female workers* | 1.33 (0.75-2.35) | 0.336 | 1.11 (0.71-1.74) | 0.633 | 0.98 (0.58-1.65) | 0.946 | 0.65 (0.29-1.48) | 0.305 | 1.82 (1.08-3.07) | 0.024 | 1.13 (0.60-2.12) | 0.706 |
| *51-75% female workers* | 1.27 (0.69-2.35) | 0.439 | 0.98 (0.58-1.67) | 0.953 | 1.02 (0.57-1.84) | 0.943 | 0.92 (0.45-1.87) | 0.816 | 0.97 (0.48-1.94) | 0.920 | 0.90 (0.45-1.81) | 0.776 |
| *>76% Female workers* | 0.80 (0.19-3.48) | 0.770 | 0.83 (0.32-2.15) | 0.701 | 0.66 (0.28-2.26) | 0.194 | <0.00 (0.0-0.0) | . | 0.92 (0.12-6.89) | 0.937 | 0.51 (0.07-3.85) | 0.514 |

*PR=Prevalence Ratio; Ref =Reference category; CI =confidence interval; Model 2: Adjusts for age, education, and ethnicity; . groups too small to calculate estimate*

*Supplementary table 11: Associations between gender-related characteristics and eGFR<60ml/min/1.73m^2^ in men across ethnic groups*

|  | Dutch | | South-Asian Surinamese | | African Surinamese | | Ghanaian | | Turkish | | Moroccan | |
| --- | --- | --- | --- | --- | --- | --- | --- | --- | --- | --- | --- | --- |
|  | PR | p-value | PR | p-value | PR | p-value | PR | p-value | PR | p-value | PR | p-value |
| **eGFR<60ml/min/1.73m^2^** | | | | | | | | | | | | |
| **Time spent on housework** | | | | | | | | | | | | |
| *0-3 hours/week* | 1.18 (0.51-2.72) | 0.707 | 1.02 (0.42-2.47) | 0.967 | 0.58 (0.21-1.60) | 0.295 | 0.60 (0.18-1.95) | 0.392 | 0.83 (0.16-4.39) | 0.826 | 1.75 (0.36-8.47) | 0.489 |
| *3-7.75 hours/week* | Ref |  | Ref |  | Ref |  | Ref |  | Ref |  | Ref |  |
| *7.75-16 hours/week* | 0.76 (0.30-1.88) | 0.549 | 0.72 (0.27-1.92) | 0.507 | 0.56 (0.21-1.41) | 0.217 | 0.74 (0.18-3.12) | 0.686 | 1.49 (0.21-10.55) | 0.693 | <0.00 (0.0-0.0) | 1.000 |
| *>16 hours/week* | 1.14 (0.39-3.29) | 0.816 | 1.08 (0.40-2.93) | 0.874 | 0.36 (0.10-1.31) | 0.121 | 0.52 (0.06-4.46) | 0.550 | 2.51 (0.35-18.07) | 0.361 | <0.00 (0.0-0.0) | . |
| **Primary earner status** | | | | | | | | | | | | |
| *Yes* | Ref |  | Ref |  | Ref |  | Ref |  | Ref |  | Ref |  |
| *No, equal income* | 1.07 (0.44-2.61) | 0.874 | 0.62 (0.19-2.02) | 0.424 | 0.32 (0.4-2.39) | 0.268 | <0.001 (0.0-0.0) | . | 0.82 (0.11-6.43) | 0.850 | 1.010 (0.14-8.80) | 0.931 |
| *No* | 0.50 (0.07-3.7) | 0.498 | 1.12 (0.62-4.75) | 0.881 | 1.91 (0.66-5.59) | 0.236 | 2.44 (0.52-11.50) | 0.261 | <0.001 (0.0-0.0) | . | <0.001 (0.0-0.0) | . |
| **Employment Status** | | | | | | | | | | | | |
| *>32 hours/week* | Ref |  | Ref |  | Ref |  | Ref |  | Ref |  | Ref |  |
| *12-32 hours/week* | 0.75 (0.16-3.52) | 0.720 | 2.24 (0.76-6.62) | 0.146 | 0.80 (0.10-6.52) | 0.834 | 0.84 (0.10-6.95) | 0.868 | <0.00 (0.0-0.0) | 1.000 | <0.00 (0.0-0.0) | 1.000 |
| *<12 hours/week incl. Homemakers* | 0.81 (0.10-6.79) | 0.849 | <00 (0.0-0.0) | . | <0.00 (0.0-0.0) | . | <0.001´(0.0-0.0) | . | <0.00 (0.0-0.0) | . | <0.00 (0.0-0.0) | . |
| *Not employed* | 1.32 (0.52-3.33) | 0.562 | 0.86 (0.36-2.08) | 0.737 | 1.93 (0.75-5.02) | 0.175 | 2.41 (0.82-7.12) | 0.111 | 2.62 (0.60-11.38) | 0.199 | 1.60 (0.35-7.42) | 0.546 |
| **Female-/Male dominated Occupation** | | | | | | | | | | | | |
| *<25% female workers* | Ref |  | Ref |  | Ref |  | Ref |  | Ref |  | Ref |  |
| *26-50% female workers* | 1.37 (0.59-3.18) | 0.461 | 1.13 (0.48-2.68) | 0.786 | 0.75 (0.23-2.39) | 0.622 | 0.59 (0.12-2.92) | 0.516 | 0.53 (0.10-2.92) | 0.467 | 1.46 (0.24-8.77) | 0.681 |
| *51-75% female workers* | 1.24 (0.50-3.05) | 0.642 | 1.01 (0.38-2.69) | 0.991 | 1.96 (0.76-5.02) | 0.162 | 0.85 (0.22-3.30) | 0.814 | 0.71 (0.13-4.03) | 0.700 | 1.04 (0.15-7.49) | 0.966 |
| *>76% Female workers* | 1.04 (0.13-8.40) | 0.969 | 1.36 (0.35-5.30) | 0.656 | 1.62 (0.35-7.64) | 0.540 | <0.00 (0.0-0.0) | . | <0.00 (0.0-0.0) | . | <0.00 (0.0-0.0) | . |

*PR=Prevalence Ratio; Ref =Reference category; CI =confidence interval; Model 2: Adjusts for age, education, and ethnicity; . groups too small to calculate estimate*

*Supplementary table 12: Associations between gender-related characteristics and ACR ≥3mg/mml in men across ethnic groups*

|  | Dutch | | South-Asian Surinamese | | African Surinamese | | Ghanaian | | Turkish | | Moroccan | |
| --- | --- | --- | --- | --- | --- | --- | --- | --- | --- | --- | --- | --- |
|  | PR | p-value | PR | p-value | PR | p-value | PR | p-value | PR | p-value | PR | p-value |
| **ACR ≥3mg/mml** | | | | | | | | | | | | |
| **Time spent on housework** | | | | | | | | | | | | |
| *0-3 hours/week* | 0.90 (0.46-1.78) | 0.770 | 1.22 (0.73-2.05) | 0.448 | 0.79 (0.42-1.49) | 0.473 | 1.20 (0.55-2.61) | 0.655 | 1.20 (0.63-2.26) | 0.583 | 1.50 (0.77-2.90) | 0.230 |
| *3-7.75 hours/week* | Ref |  | Ref |  | Ref |  | Ref |  | Ref |  | Ref |  |
| *7.75-16 hours/week* | 0.78 (0.38-1.61) | 0.497 | 0.94 (0.53-1.66) | 0.831 | 0.86 (0.48-1.54) | 0.610 | 1.34 (0.54-3.29) | 0.527 | 1.01 (0.41-2.48) | 0.977 | 0.62 (0.22-1.75) | 0.366 |
| *>16 hours/week* | 0.90 (0.33-2.44) | 0.840 | 1.38 (0.77-2.47) | 0.276 | 0.88 (0.44.1.75) | 0.706 | 1.28 (0.43-3.82) | 0.663 | 1.18 (0.42-3.36) | 0.757 | 2.30 (0.97-5.46) | 0.060 |
| **Primary earner status** | | | | | | | | | | | | |
| *Yes* | Ref |  | Ref |  | Ref |  | Ref |  | Ref |  | Ref |  |
| *No, equal income* | 0.68 (0.30-1.53) | 0.354 | 0.49 (0.24-1.00) | 0.050 | 0.62 (0.27-1.44) | 0.267 | 2.08 (1.03-4.22) | 0.042 | 1.03 (0.49-2.16) | 0.937 | 0.35 (0.09-1.44) | 0.146 |
| *No* | 0.90 (0.32-2.56) | 0.845 | 0.67 (0.29-1.57) | 0.356 | 0.80 (0.36-1.80) | 0.590 | 1.81 (0.63-5.16) | 0.269 | 0.97 (0.38-2.49) | 0.949 | 2.11 (0.98-4.53) | 0.056 |
| **Employment Status** | | | | | | | | | | | | |
| *>32 hours/week* | Ref |  | Ref |  | Ref |  | Ref |  | Ref |  | Ref |  |
| *12-32 hours/week* | 0.98 (0.37-2.57) | 0.961 | 0.80 (0.32-2.03) | 0.638 | 1.23 (0.54-2.78) | 0.628 | 1.63 (0.69-3.85) | 0.265 | 1.50 (0.70-3.22) | 0.299 | 0.74 (0.22-2.47) | 0.627 |
| *<12 hours/week incl. Homemakers* | 0.72 (0.10-5.38) | 0.749 | <.00 (0.0-0.0) | . | 0.83 (0.11-6.10) | 0.857 | 1.10 (0.15-8.85) | 0.928 | <.00 (0.0-0.0) | . | <.00 (0.0-0.0) | . |
| *Not employed* | 1.30 (0.66-2.55) | 0.442 | 1.41 (0.91-2.17) | 0.125 | 1.30 (0.78-2.15) | 0.316 | 1.38 (0.71-2.69) | 0.344 | 1.16 (0.70-1.92) | 0.571 | 1.69 (0.97-2.94) | 0.064 |
| **Female-/Male dominated Occupation** | | | | | | | | | | | | |
| *<25% female workers* | Ref |  | Ref |  | Ref |  | Ref |  | Ref |  | Ref |  |
| *26-50% female workers* | 1.18 (0.57-2.42) | 0.817 | 1.16 (0.72-1.87) | 0.257 | 1.07 (0.60-1.88) | 0.947 | . | . | 2.01 (1.16-3.49) | 0.013 | 1.26 (0.66-2.40) | 0.578 |
| *51-75% female workers* | 1.28 (0.60-2.74) | 0.900 | 0.97 (0.54-1.73) | 0.161 | 1.05 (0.54-2.02) | 0.922 | . | . | 1.03 (0.49-2.15) | 0.540 | 1.00 (0.49-2.05) | 0.435 |
| *>76% Female workers* | 1.07 (0.24-4.79) | 0.931 | 0.42 (0.10-1.76) | 0.233 | 1.01 (0.35-2.93) | 0.987 | . | . | 1.08 (0.14-8.10) | 0.942 | 0.56 (0.07-4.27) | 0.578 |

*PR=Prevalence Ratio; Ref =Reference category; CI =confidence interval; Model 2: Adjusts for age, education, and ethnicity; . groups too small to calculate estimate*

Supplementary table 13: Population attributable fraction in the total population

|  | | **PR (95% CI)** | **pd** | **PAF** |
| --- | --- | --- | --- | --- |
| **Women CKD (CKD-EPI 2012)** | | | | |
| **Housework *-*** *a lot (unexposed) vs a little (exposed)* | Model 2 | 1.14 (0.94-1.37) | 0.172 | 0.21 |
|  | Model 3 | 1.12 (0.93-1.35) | 0.172 | 0.18 |
| **Employment Status *-*** *full time (unexposed) vs part time/not working (exposed)* | Model 2 | 1.49 (1.23-1.80) | 0.808 | 0.265 |
|  | Model 3 | 1.37 (1.13-1.67) | 0.808 | 0.218 |
| **Men CKD (CKD-EPI 2012)** | | | | |
| **Primary Earner Status ***** | Model 2 | 1.42 (1.05-1.92) | 0.810 | 0.375 |
|  |  | 1.63 (1.07-2.46) | 0.091 |  |
|  | Model 3 | 1.33 (0.98-1.81) | 0.810 | 0.336 |
|  |  | 1.59 (1.05-2.42) | 0.091 |  |
| **Employment Status *-*** *full time (Unexposed) vs part time/not working (exposed)* | Model 2 | 1.32 (1.09-1.59) | 0.579 | 0.14 |
|  | Model 3 | 1.27 (1.04-1.54) | 0.579 | 0.121 |
| **Women eGFR <60ml/min/1.73m^2^** | | | | |
| **Primary Earner Status***** | Model 2 | 1.73 (1.08-2.78) | 0.649 | 0.528 |
|  |  | 1.88 (1.05-3.37) | 0.183 |  |
|  | Model 3 | 1.61 (1.0-2.59) | 0.649 | 0.499 |
|  |  | 1.89 (1.06-3.39) | 0.183 |  |
| **Men eGFR <60ml/min/1.73m^2^** | | | | |
| **Employment Status *-*** *full time (unexposed) vs part time/not working (exposed)* | Model 2 | 1.45 (0.96-2.19) | 0.702 | 0.216 |
|  | Model 3 | 1.37 (0.90-2.08) | 0.702 | 0.188 |
| **Women ACR ≥3mg/mml** | | | | |
| **Employment status *-*** *full time (unexposed) vs part time/not working (exposed)* | Model 2 | 1.4 (1.14-1.71) | 0.803 | 0.228 |
|  | Model 3 | 1.28 (1.04-1.57) | 0.803 | 0.173 |
| **Men ACR ≥3mg/mml** | | | | |
| **Employment status *-*** *full time (unexposed) vs part time/not working (exposed)* | Model 2 | 1.29 (1.06-1.59) | 0.558 | 0.127 |
|  | Model 3 | 1.24 (1.01-1.53) | 0.558 | 0.109 |

*PR (Prevalence Ratio) 95% CI (Confidence Interval); Pd (the proportion of cases "exposed" to the risk factor); PAF (Population Attributable Fraction); * Dichotomisation of variable categories not possible, therefore multi-categorical estimation; Model 2: Adjusted for Age, education, and ethnicity; Model 3: Model 2 + traditional risk factors (Hypertension, Diabetes Mellitus, Hypercholesterolemia, BMI, Smoking Status)*

*Supplementary table 14: Population attributable fractions across ethnic groups*

|  | Dutch | | | South Asian Surinamese | | | African Surinamese | | | Ghanaian | | | Turkish | | | Moroccan | | |
| --- | --- | --- | --- | --- | --- | --- | --- | --- | --- | --- | --- | --- | --- | --- | --- | --- | --- | --- |
|  | PR | pd | PAF (%) | PR | pd | PAF (%) | PR | pd | PAF (%) | PR | pd | PAF (%) | PR | pd | PAF (%) | PR | pd | PAF (%) |
| **Women CKD (CKD-EPI 2012)** | | | | | | | | | | | | | | | | | | |
| **Time spent on Housework** | | | | | | | | | | | | | | | | | | |
| Model 2 | 1.14 (0.94-1.37) | 0.123 | 1.5 | 1.14 (0.94-1.37) | 0.208 | 2.5 | 1.14 (0.94-1.37) | 0.184 | 2.2 | 1.14 (0.94-1.37) | 0.216 | 2.6 | 1.14 (0.94-1.37) | 0.169 | 2 | 1.14 (0.94-1.37) | 0.127 | 1.5 |
| Model 3 | 1.12 (0.93-1.35) | 0.123 | 1.3 | 1.12 (0.93-1.35) | 0.208 | 2.2 | 1.12 (0.93-1.35) | 0.184 | 1.9 | 1.12 (0.93-1.35) | 0.216 | 2.3 | 1.12 (0.93-1.35) | 0.169 | 1.7 | 1.12 (0.93-1.35) | 0.127 | 1.3 |
| **Employment Status** | | | | | | | | | | | | | | | | | | |
| Model 2 | 1.49 (1.23-1.80) | 0.686 | 22.5 | 1.49 (1.23-1.80) | 0.791 | 25.9 | 1.49 (1.23-1.80) | 0.729 | 23.9 | 1.49 (1.23-1.80) | 0.754 | 24.7 | 1.49 (1.23-1.80) | 0.928 | 30.4 | 1.49 (1.23-1.80) | 0.891 | 29.2 |
| Model 3 | 1.37 (1.13-1.67) | 0.686 | 18.5 | 1.37 (1.13-1.67) | 0.791 | 21.3 | 1.37 (1.13-1.67) | 0.729 | 19.6 | 1.37 (1.13-1.67) | 0.754 | 20.4 | 1.37 (1.13-1.67) | 0.928 | 25.0 | 1.37 (1.13-1.67) | 0.891 | 24.1 |
| **Men CKD (CKD-EPI 2012)** | | | | | | | | | | | | | | | | | | |
| Primary Earner Status | | | | | | | | | | | | | | | | | | |
| Model 2 | 1.42 (1.05-1.92) | 0.795 | 40.4 | 1.42 (1.05-1.92) | 0.869 | 35.4 | 1.42 (1.05-1.92) | 0.809 | 36.3 | 1.42 (1.05-1.92) | 0.685 | 45.1 | 1.42 (1.05-1.92) | 0.827 | 37.4 | 1.42 (1.05-1.92) | 0.803 | 33.5 |
|  | 1.63 (1.07-2.46) | 0.064 |  | 1.63 (1.07-2.46) | 0.057 |  | 1.63 (1.07-2.46) | 0.112 |  | 1.63 (1.07-2.46) | 0.111 |  | 1.63 (1.07-2.46) | 0.074 |  | 1.63 (1.07-2.46) | 0.164 |  |
| Model 3 | 1.33 (0.98-1.81) | 0.795 | 36.4 | 1.33 (0.98-1.81) | 0.869 | 31.3 | 1.33 (0.98-1.81) | 0.809 | 32.4 | 1.33 (0.98-1.81) | 0.685 | 41.8 | 1.33 (0.98-1.81) | 0.827 | 33.4 | 1.33 (0.98-1.81) | 0.803 | 29.5 |
|  | 1.59 (1.05-2.42) | 0.064 |  | 1.59 (1.05-2.42) | 0.057 |  | 1.59 (1.05-2.42) | 0.112 |  | 1.59 (1.05-2.42) | 0.111 |  | 1.59 (1.05-2.42) | 0.074 |  | 1.59 (1.05-2.42) | 0.164 |  |
| **Employment Status** | | | | | | | | | | | | | | | | | | |
| Model 2 | 1.32 (1.09-1.59) | 0.620 | 15 | 1.32 (1.09-1.59) | 0.618 | 15 | 1.32 (1.09-1.59) | 0.567 | 13.7 | 1.32 (1.09-1.59) | 0.537 | 13 | 1.32 (1.09-1.59) | 0.531 | 12.9 | 1.32 (1.09-1.59) | 0.565 | 13.7 |
| Model 3 | 1.27 (1.04-1.54) | 0.620 | 12.9 | 1.27 (1.04-1.54) | 0.618 | 12.9 | 1.27 (1.04-1.54) | 0.567 | 11.8 | 1.27 (1.04-1.54) | 0.537 | 11.2 | 1.27 (1.04-1.54) | 0.531 | 11.1 | 1.27 (1.04-1.54) | 0.565 | 11.8 |
| **Women eGFR <60ml/min/1.73m^2^** | | | | | | | | | | | | | | | | | | |
| **Primary Earner Status** | | | | | | | | | | | | | | | | | | |
| Model 2 | 1.73 (1.08-2.78) | 0.586 | 53.3 | 1.73 (1.08-2.78) | 0.514 | 56.7 | 1.73 (1.08-2.78) | 0.821 | 46.8 | 1.73 (1.08-2.78) | 0.813 | 46.4 | 1.73 (1.08-2.78) | 0.600 | 54.7 | 1.73 (1.08-2.78) | 0.615 | 60.3 |
|  | 1.88 (1.05-3.37) | 0.241 |  | 1.88 (1.05-3.37) | 0.257 |  | 1.88 (1.05-3.37) | 0.107 |  | 1.88 (1.05-3.37) | 0.125 |  | 1.88 (1.05-3.37) | 0.200 |  | 1.88 (1.05-3.37) | 0.077 |  |
| Model 3 | 1.61 (0.99-2.59) | 0.586 | 49.2 | 1.61 (0.99-2.59) | 0.514 | 54.4 | 1.61 (0.99-2.59) | 0.821 | 43.4 | 1.61 (0.99-2.59) | 0.813 | 42.8 | 1.61 (0.99-2.59) | 0.600 | 52.1 | 1.61 (0.99-2.59) | 0.615 | 56.9 |
|  | 1.89 (1.06-3.39) | 0.241 |  | 1.89 (1.06-3.39) | 0.257 |  | 1.89 (1.06-3.39) | 0.107 |  | 1.89 (1.06-3.39) | 0.125 |  | 1.89 (1.06-3.39) | 0.200 |  | 1.89 (1.06-3.39) | 0.077 |  |
| **Men CKD eGFR <60ml/min/1.73m^2^** | | | | | | | | | | | | | | | | | | |
| **Employment Status** | | | | | | | | | | | | | | | | | | |
| Model 2 | 1.45 (0.96-2.19) | 0.743 | 22.9 | 1.45 (0.96-2.19) | 0.676 | 20.8 | 1.45 (0.96-2.19) | 0.741 | 22.8 | 1.45 (0.96-2.19) | 0.600 | 18.5 | 1.45 (0.96-2.19) | 0.727 | 22.4 | 1.45 (0.96-2.19) | 0.667 | 20.5 |
| Model 3 | 1.37 (0.90-2.08) | 0.743 | 19.9 | 1.37 (0.90-2.08) | 0.676 | 18.1 | 1.37 (0.90-2.08) | 0.741 | 19.8 | 1.37 (0.90-2.08) | 0.600 | 16.1 | 1.37 (0.90-2.08) | 0.727 | 19.4 | 1.37 (0.90-2.08) | 0.667 | 17.8 |
| **Women ACR ≥3mg/mml** | | | | | | | | | | | | | | | | | | |
| **Employment Status** | | | | | | | | | | | | | | | | | | |
| Model 2 | 1.40 (1.14-1.71) | 0.679 | 19.2 | 1.40 (1.14-1.71) | 0.757 | 21.4 | 1.40 (1.14-1.71) | 0.717 | 20.3 | 1.40 (1.14-1.71) | 0.743 | 21 | 1.40 (1.14-1.71) | 0.924 | 26.1 | 1.40 (1.14-1.71) | 0.887 | 25.1 |
| Model 3 | 1.28 (1.04-1.57) | 0.679 | 14.6 | 1.28 (1.04-1.57) | 0.757 | 16.2 | 1.28 (1.04-1.57) | 0.717 | 15.4 | 1.28 (1.04-1.57) | 0.743 | 16 | 1.28 (1.04-1.57) | 0.924 | 19.9 | 1.28 (1.04-1.57) | 0.887 | 19.1 |
| **Men ACR ≥3mg/mml** | | | | | | | | | | | | | | | | | | |
| **Employment Status** | | | | | | | | | | | | | | | | | | |
| Model 2 | 1.29 (1.06-1.59) | 0.538 | 12.2 | 1.29 (1.06-1.59) | 0.594 | 13.4 | 1.29 (1.06-1.59) | 0.579 | 13.1 | 1.29 (1.06-1.59) | 0.523 | 11.8 | 1.29 (1.06-1.59) | 0.50 | 11.3 | 1.29 (1.06-1.59) | 0.583 | 13.2 |
| Model 3 | 1.24 (1.01-1.53) | 0.538 | 10.5 | 1.24 (1.01-1.53) | 0.594 | 11.6 | 1.24 (1.01-1.53) | 0.579 | 11.3 | 1.24 (1.01-1.53) | 0.523 | 10.3 | 1.24 (1.01-1.53) | 0.50 | 9.8 | 1.24 (1.01-1.53) | 0.583 | 11.4 |

*PR (Prevalence Ratio) 95% CI (Confidence Interval); Pd (the proportion of cases "exposed" to the risk factor); PAF (Population Attributable Fraction); * Dichotomisation of variable categories not possible, therefore multi-categorical estimation; Model 2: Adjusted for Age, education, and ethnicity; Model 3: Model 2 + traditional risk factors (Hypertension, Diabetes Mellitus, Hypercholesterolemia, BMI, Smoking Status)*

**References**

1. Solera, C., & Mencarini, L. (2018). The gender division of housework after the first child: a comparison among Bulgaria, France and the Netherlands. Community, Work & Family, 21(5), 519-540. doi:10.1080/13668803.2018.1528969
2. Kemppainen LM, Kemppainen TT, Reippainen JA, Salmenniemi ST, Vuolanto PH. Use of complementary and alternative medicine in Europe: Health-related and sociodemographic determinants. Scand J Public Health. Jun 2018;46(4):448-455. doi:10.1177/1403494817733869
3. Kataria A, Trasande L, Trachtman H. The effects of environmental chemicals on renal function. Nat Rev Nephrol. Oct 2015;11(10):610-25. doi:10.1038/nrneph.2015.94
4. Nauffal M, Gabardi S. Nephrotoxicity of Natural Products. Blood Purif. 2016;41(1-3):123-9. doi:10.1159/000441268
5. CBS (2021). Labour participation; position in the household. Accessed 27 March 2023. <https://opendata.cbs.nl/statline/#/CBS/en/dataset/82956ENG/table?ts=1650367555078>
6. Vart, P., Gansevoort, R. T., Coresh, J., Reijneveld, S. A., & Bültmann, U. (2013). Socioeconomic measures and CKD in the United States and The Netherlands. Clin J Am Soc Nephrol, 8(10), 1685-1693. doi:10.2215/cjn.12521212
